# Transmission of Hypervirulence Traits via Sexual Reproduction within and between Lineages of the Human Fungal Pathogen *Cryptococcus gattii*

**DOI:** 10.1371/journal.pgen.1003771

**Published:** 2013-09-05

**Authors:** Kerstin Voelz, Hansong Ma, Sujal Phadke, Edmond J. Byrnes, Pinkuan Zhu, Olaf Mueller, Rhys A. Farrer, Daniel A. Henk, Yonathan Lewit, Yen-Ping Hsueh, Matthew C. Fisher, Alexander Idnurm, Joseph Heitman, Robin C. May

**Affiliations:** 1 Institute of Microbiology and Infection & School of Biosciences, University of Birmingham, Birmingham, United Kingdom; 2 The National Institute of Health Research Surgical Reconstruction and Microbiology Research Centre, Queen Elizabeth Hospital Birmingham, Birmingham, United Kingdom; 3 Department of Molecular Genetics and Microbiology, Duke University, Durham, North Carolina, United States of America; 4 School of Biological Sciences, University of Missouri, Kansas City, Missouri, United States of America; 5 Department of Infectious Disease Epidemiology, School of Public Health, Imperial College London, London, United Kingdom; University College Dublin, Ireland

## Abstract

Since 1999 a lineage of the pathogen *Cryptococcus gattii* has been infecting humans and other animals in Canada and the Pacific Northwest of the USA. It is now the largest outbreak of a life-threatening fungal infection in a healthy population in recorded history. The high virulence of outbreak strains is closely linked to the ability of the pathogen to undergo rapid mitochondrial tubularisation and proliferation following engulfment by host phagocytes. Most outbreaks spread by geographic expansion across suitable niches, but it is known that genetic re-assortment and hybridisation can also lead to rapid range and host expansion. In the context of *C. gattii*, however, the likelihood of virulence traits associated with the outbreak lineages spreading to other lineages via genetic exchange is currently unknown. Here we address this question by conducting outgroup crosses between distantly related *C. gattii* lineages (VGII and VGIII) and ingroup crosses between isolates from the same molecular type (VGII). Systematic phenotypic characterisation shows that virulence traits are transmitted to outgroups infrequently, but readily inherited during ingroup crosses. In addition, we observed higher levels of biparental (as opposed to uniparental) mitochondrial inheritance during VGII ingroup sexual mating in this species and provide evidence for mitochondrial recombination following mating. Taken together, our data suggest that hypervirulence can spread among the *C. gattii* lineages VGII and VGIII, potentially creating novel hypervirulent genotypes, and that current models of uniparental mitochondrial inheritance in the *Cryptococcus* genus may not be universal.

## Author Summary

How infections spread within the human population is an important question in forecasting potential epidemics. One way to investigate potential mechanisms is to test experimentally whether combinations of genes that confer high virulence are able to spread to less-virulent lineages. Here, we address this question in a fungal pathogen that is causing an outbreak of meningitis in healthy humans in Canada and the Pacific Northwest. We demonstrate that virulence traits are easily transmitted between closely related pathogenic strains, but are more difficult to transmit to more distant lineages. In addition, we show that a paradigm of organelle inheritance, namely that mitochondria are inherited uniparentally from the **a** mating type, is altered in the R265α outbreak strain such that it transmits its mitochondrial genome to 25–30% of its progeny. This biparental inheritance likely contributes to increased mitochondrial recombination. Taken together, our data suggest that virulence traits may be relatively mobile within this species and that current models of mitochondrial inheritance may require revising.

## Introduction

*Cryptococcus neoformans* and *C. gattii* are the causative agents of cryptococcosis in humans. *C. neoformans* typically infects HIV-infected individuals and other patients with immunodeficiencies, but has also been found in apparently immunocompetent individuals in the Far East [1], [2]. *C. gattii* is a primary pathogen that causes disease in otherwise healthy people [3], [4], but has also been found in HIV patients in Malawi, Africa and California, USA [5], [6]. *C. gattii* accounts for less than 1% of all cryptococcosis cases, and until the late 1990s occurred mostly in subtropical regions of the world. However, in 1999, an outbreak of *C. gattii* was reported on Vancouver Island in domestic pets and people [7]–[9]. This outbreak spread to mainland Canada and then into the northwestern states of the United States [10]–[13] and currently numbers more than 400 cases [14]–[17].

*C. gattii* is divided into distinct clades (VGI-VGIV) [14], with the outbreak originating on Vancouver Island, and a more recent outbreak in Oregon [18], , being caused by three clonal groups within VGII (VGIIa, VGIIb and VGIIc) [20]. These hypervirulent outbreaks are characterized by an unusual ability of the pathogen to parasitise host phagocytic cells: upon engulfment by macrophages, outbreak strains initiate mitochondrial tubularisation and rapid intracellular proliferation of the fungus [21].

Cryptococcosis is not spread from infected animals or humans to susceptible hosts but rather infections are acquired from the environment. Hence, cryptococcal species likely experience strong selective pressure from factors encountered within environmental niches. Genetic recombination by meiotic sexual reproduction in eukaryotic pathogens is a widely-occurring mechanism that generates genetic diversity (and hence novel phenotypic diversity) but carries the risk of destroying beneficial gene combinations [22]. The genetic distance across which genetic recombination occurs yields very different outcomes. Outcrossing and hybridization can result in dramatic changes to genotype and resulting virulence phenotypes. For example, Grigg *et al.* [23] have demonstrated that outcrossing sexual recombination can be a major force in shaping eukaryotic pathogens, since recombinant *Toxoplasma* progeny from crosses between two distinct ancestral lines type II and type III are significantly more virulent than either parent. A similar hypothesis has been proposed for the origin of *C. gattii* outbreak strains [24]. However, outcrossing can also come at the cost of breaking up highly-fit coadapted gene-complexes, such as those that enable host adaptation [25], [26], and can result in lethal levels of genetic load resulting in widespread inviability.

Therefore, estimating how likely it is for hypervirulence traits to move between *C. gattii* lineages by recombination is critical both for predicting the likelihood of novel hypervirulent genotypes occurring and, more broadly, in understanding the origins of infectious outbreaks. In addition, given that the expression of mitochondrially-encoded genes correlates with virulence in *C. gattii*, but mitochondrial genes do not contribute to virulence in *C. neoformans* [21], [27], important questions remain about the relative role of mitochondrially-encoded, versus nuclear-encoded, genes in controlling virulence in this pathogen. Here we address both of these questions via a series of genetic crosses, followed by comprehensive phenotypic analyses. Our findings demonstrate that hypervirulence in *C. gattii* is a complex, multigenic trait. Surprisingly, however, this trait can be transmitted relatively easily to other lineages and is not strictly limited to one mitochondrial genotype. Finally, we show that, in contrast to existing paradigms, mitochondrial inheritance in *C. gattii* is not strictly uniparental and thus current models of genetic exchange in this pathogenic clade should be revisited.

## Results

### Experimental Design of Ingroup and Outgroup Crosses

This study addresses two questions:

1. How likely is the spread of hypervirulence traits between *C. gattii* lineages?
2. What is the contribution of the mitochondrial genome in controlling virulence in *C. gattii*?

**Figure 1:**
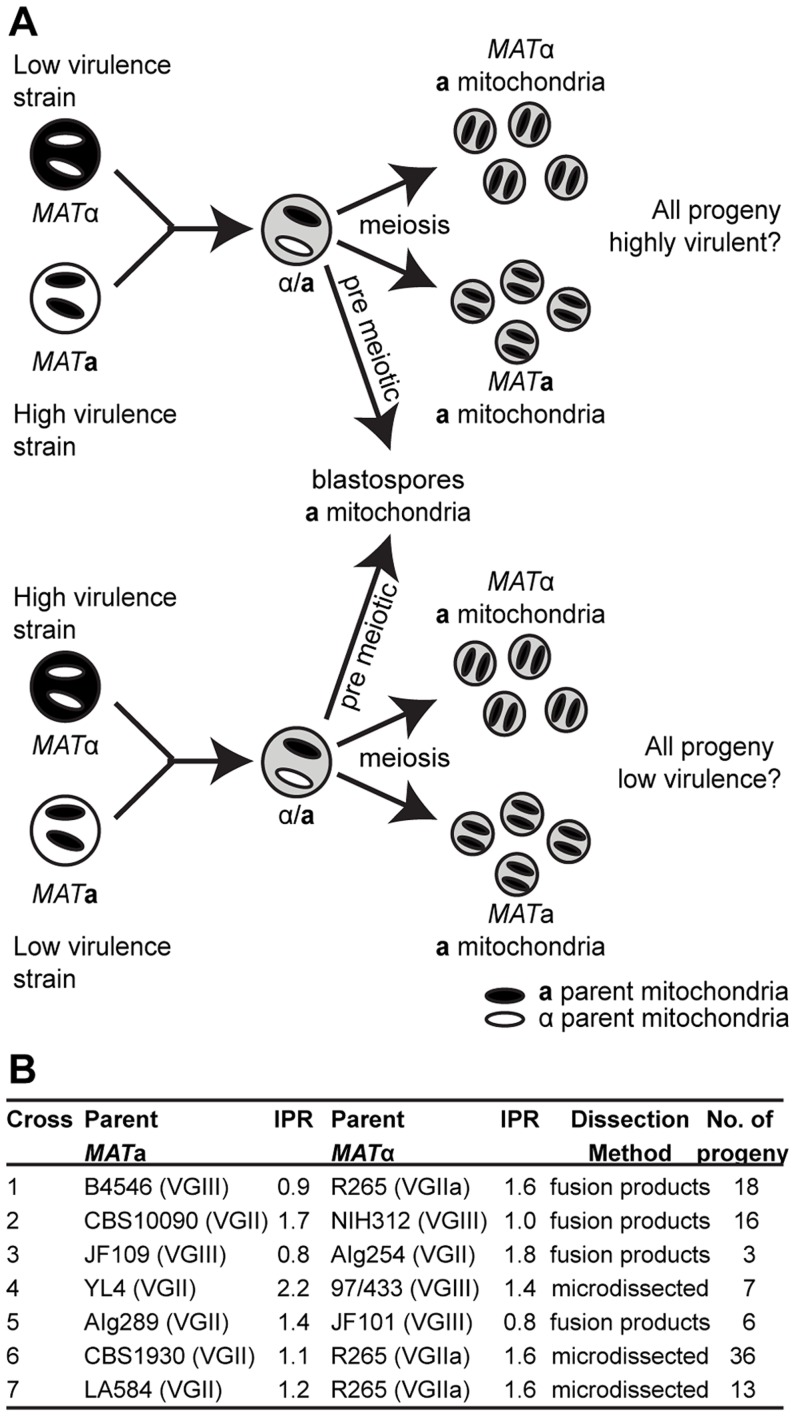
Experimental design of ingroup and outgroup crosses. **A**) Schematic illustration of the crosses between a low or highly virulent α parent and a high or low virulence **a** parent respectively. **B**) Summary of the strains used for mating, their ability to proliferate intracellularly within macrophages (IPR), and the number of progeny isolated from each cross. ^†^Subsequent whole genome sequence analysis revealed that the progeny and restored ‘haploid’ strains are aneuploid strains. Progeny showed regions of triploidy and restored strains regions of diploidy (Figure S1).

Recent work has demonstrated that the ability to change mitochondrial morphology is closely linked to intracellular proliferation and thus hypervirulence in *C. gattii* [21]. In the related pathogen *C. neoformans*, as in most eukaryotes, mitochondria are inherited from only one parent (in this case the *MAT* **a** parent) following mating [28], [29]. To exploit this uniparental inheritance and to test the likelihood of virulence traits spreading within the *C. gattii* population, we conducted a series of crosses in which progeny would inherit mitochondria either from a hypervirulent parent, or from a non-outbreak strain exhibiting wild type virulence. If phenotypes associated with hypervirulence (mitochondrial tubularisation in response to phagocytosis and rapid intracellular proliferation) were solely determined by mitochondrial genotypes then all progeny from each cross would have the same phenotype as the *MAT* **a** parent (Figure 1A).

The ability to proliferate within macrophages is a proven predictor of virulence in *C. neoformans* and *C. gattii* [5], [21]. To assess virulence in a comprehensive progeny set in this study, we utilized intracellular proliferation as a proxy-measure of virulence and investigated its relationship to mitochondrial tubularisation.

Our experimental approach included both outgroup crosses, between strains from two different molecular groups (VGII and VGIII), and ingroup crosses, between strains from the same molecular group (VGII) (Figure 1B). Despite the fact that VGIII strains are more fertile than other *C. gattii* strains [24], [30], [31], experimental mating of *C. gattii* strains is extremely difficult in the laboratory setting. However, within the VGIII lineage, the VGIII pair B4546 (*MAT* **a**) and NIH312 (*MAT*α) had previously been identified as mating test strains in an extensive screening study [32]. These strains also exhibit low intracellular proliferation rates and hence were chosen for the outgroup crosses in this study. Disappointingly, after various attempts, we and others were unable to mate *MAT* **a**-VGII with *MAT*α–VGII strains that exhibit explicitly distinct intracellular proliferation values. We were, however, able to conduct mating between VGII strains with more similar intracellular proliferation rates allowing for dissection of individual spores.

Overall, there was a low rate of spore germination in both the VGII **a** x VGIII α and VGII α x VGIII **a** mating pairs. This is consistent with population genetic evidence [24], [33] that these molecular types are genetically isolated with respect to nuclear DNA exchange and hence consistent with assignment as distinct species [34]. For each mating (B4546 x R265, CBS10090 x NIH312 and JF101 x AIg289), at least 50 individual spores (50, 50, 63) were dissected, and in all cases none germinated (0/163). After extensive attempts we were able to obtain six viable microdissected spores (6/140) from an outgroup cross (strains YL4 x 97/433). Crosses across species boundaries in *Escherichia*, *Salmonella* and *Saccharomyces* species are known to suffer from extensive DNA mismatches and cause serious problems during meiosis attributable to the mismatch repair system aborting homologous recombination [35]–[37]. To circumvent this substantial barrier and in an attempt to generate a more comprehensive working progeny set, a region of highly dense spores and hyphae was selected, plated, and colonies that arose were isolated and characterized. This type of analysis is therefore subject to possible isolation of parental yeast cells, blastospores (yeast cells derived from hyphae post-fusion but prior to nuclear fusion and meiosis), diploid fusion products, and true haploid meiotic progeny. For these reasons, and to better understand the dynamics of mating between these two distinct molecular types, the resulting progeny sets were subjected to molecular (MultiLocus Sequence Typing; MLST/Fluorescence-activated cell sorting; FACS) and phenotypic (self-filamentation) analyses.

As an additional approach, VGIII strains carrying the *crg1::NEO* mutation were crossed with a VGII strain carrying the *bwc2::NAT* mutation (JF101xAIg289 and JF109xAIg254) (Figure 1B). The parental strain AIg289 also carried a mutation within the *FUR1* gene that confers resistant to 5-fluorouracil. Mutation of both Crg1 and Bwc2 enhance mating under conditions that normally repress it. Basidiospores were unviable (0/63 germinated). However, putative fusion or post-meiotic strains were isolated by plating onto YPD medium containing both nourseothricin and G418. Strains were examined for phenotypes and PCR-RFLP markers.

### Molecular Characterisation of Progeny from Outgroup Cross R265 x B4546

**Figure 2:**
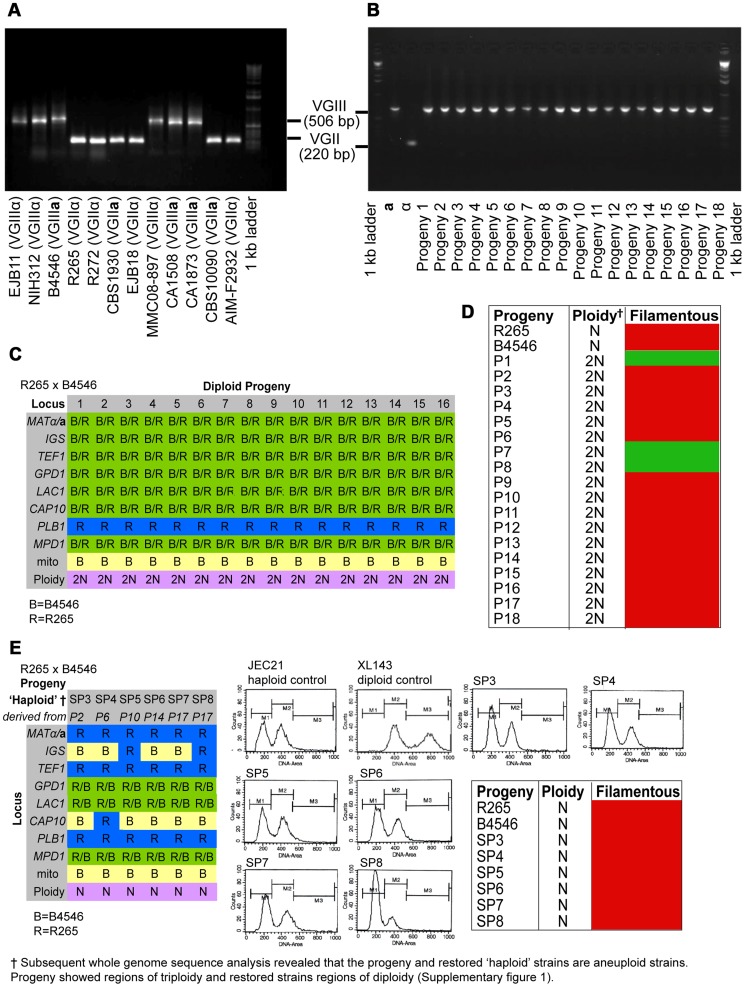
Molecular characterisation of the progeny from outgroup crosses of R265 x B4546. *ATP6* primers were used to differentiate VGII and VGIII molecular types and allow for the documentation of uniparental mitochondrial inheritance in *C. gattii*. **A**) mtDNA PCR products for 12 strains (six VGII and six VGIII) where a shorter amplicon is produced from VGII isolates compared to VGIII isolates. **B**) Uniparental inheritance of the mitochondrial genome in 18 R265 x B4656 progeny. These progeny showed the same length as their VGIII *MAT* **a** parent (isolate B4546) and not the α parental strain (isolate R265). **C**) MLST analysis of B4546 x R265 progeny was conducted at eight unlinked loci, and scored as VGIII parental (blue, B = B4546), VGII parental (yellow, R = R265), or both VGII and VGIII (green). The mitochondrial inheritance is also indicated (uniparental). The ploidy determination is listed based on FACS analysis. **D**) Self-fertility of R265 x B4546 progeny. Green coloration indicates self-fertility and red coloration indicates no signs of self-fertility after four weeks. **E**) MLST analysis of B4546 x R265 haploid progeny was conducted at eight unlinked loci, and scored as VGIII parental (blue, B = B4546) or VGII parental (yellow, R = R265). The mitochondrial inheritance is also indicated (uniparental). The ploidy determination is listed based on FACS analysis. None of the haploid progeny showed self-fertility after four weeks (indicated by red coloration).

In the mating pair between VGII α (R265) x VGIII **a** (B4546), we isolated a total of 18 progeny as described above. Amplification of the *ATP6* gene (encoded by the mitochondrial genome) revealed that 100% of the progeny inherited the **a** mitochondrial genome (Figures 2A&B and Supplementary Information), consistent with previous studies showing uniparental mitochondrial inheritance during **a**-α mating [28], [38], [39].

Based on FACS analysis, we then determined that 18/18 isolates showed signs of diploidy (Figure 2 C&D) and, in line with this, MLST analysis showed signs of heterozygosity at 7/8 markers (Figure 2C). In all of the isolates, the remaining marker (*PLB1*) specifically amplified the VGII allele, most likely due to primer bias or loss of heterozygosity caused by mitotic gene conversion or partial chromosomal loss. Interestingly, while all 18 strains retained copies of both *SXI1/SXI2*, only 17% (3/18) were self-fertile (Figure 2D).

Six progeny that were restored towards haploidy following extensive passage on YPD medium, and these showed recombinant genotypes in MLST analysis with alleles contributed by both parental strains, and ploidy was assessed by FACS analysis (Figure 2E). None of the progeny showed signs of self-filamentation, although none were derived from one of the three self-filamentous parents (Figure 2E).

It is problematic to distinguish aneuploid progeny by FACS analysis alone. MLST analysis showed signs of heterozygosity for the markers *MPD, GPD1* and *LAC1* for all restored “haploid” progeny. We therefore sequenced the genomes of four of the diploid progeny as well as the respective “haploid restored” progeny and examined read mapping coverage and variant ratios to determine the ploidy in these strains. This analysis revealed that all progeny were broadly diploid/haploid, but most also carried aneuploid regions within at least one of their chromosomes (Supplementary Information and Figure S1). This analysis also provided further information about the progeny and restored “haploid” strains' genetic background: *PLB1* was found to be monoallelic in MLST analysis for progeny and restored “haploid” strains but is heterozygous according to the genome data. *IGS* and *TEF1* are also monoallelic in the MLST analysis but biallelic according to the genomic data whereas *CAP10* is monoallelic in both MLST and genomic data, as is the *MAT* locus for which the entire chromosome is monoallelic in all strains.

Similar chromosomal abnormalities have previously been described as a common feature in *C. gattii* and *C. neoformans* and been suggested as an adaptive mechanism to stresses such as exposure to antifungals [4], [40]–[44].

### Molecular Characterisation of Progeny from Outgroup Cross CBS10090 x NIH312

**Figure 3:**
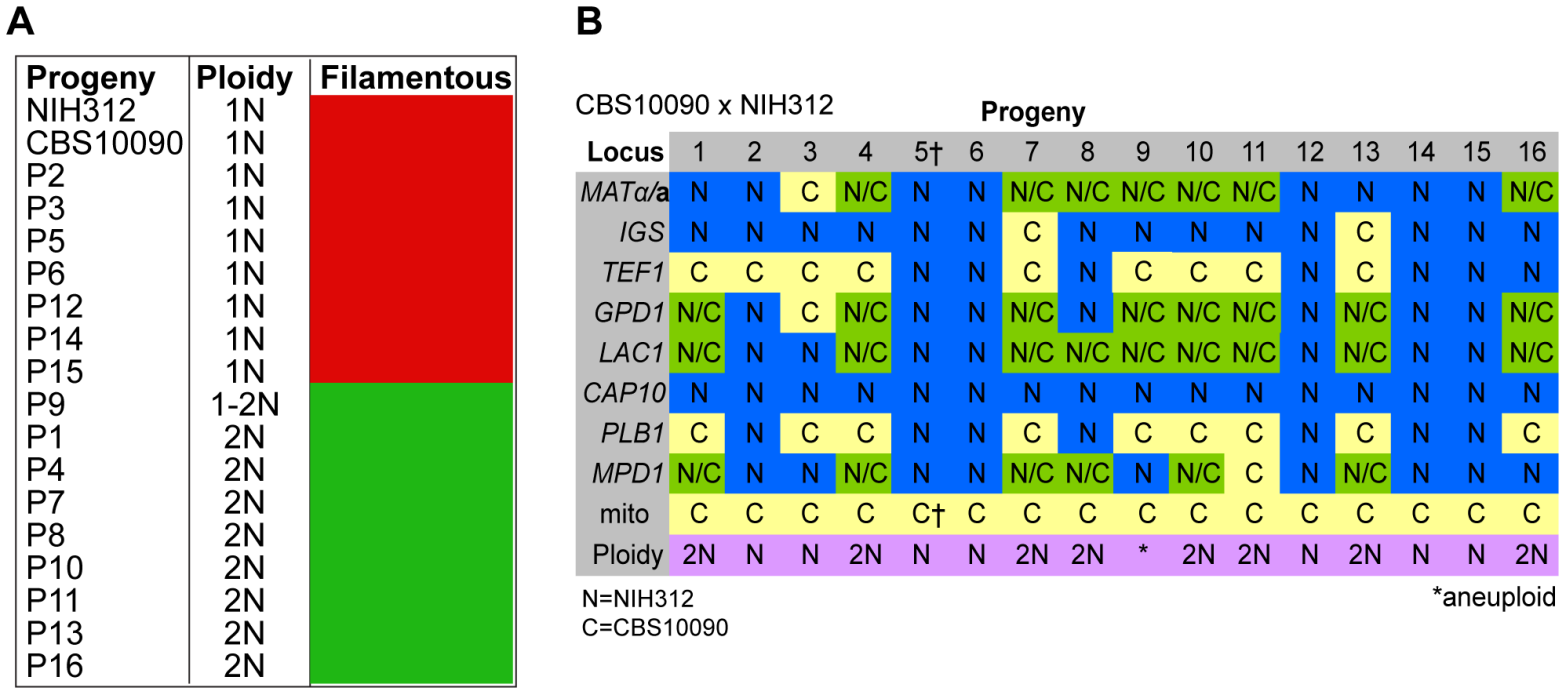
Molecular characterisation of progeny from outgroup cross CBS10090 x NIH312. **A**) Self-fertility of NIH312 x CBS10090 progeny. Green coloration indicates self-fertility and red coloration indicates no signs of self-fertility after four weeks. **B**) MLST analysis of NIH312 x CBS10090 progeny was conducted at eight unlinked loci, and scored as VGIII parental (blue), VGII parental (yellow), or both VGII and VGIII (green). The mitochondrial inheritance is also indicated (uniparental). The ploidy determination is listed based on FACS analysis. Progeny 9 is indicated as 1–2N due to an unclear FACS plot, although the molecular and self-fertility assays indicate it is diploid. ^†^CBS10090 mitochondrial type by MLST, but subsequent sequencing indicated that it actually carries a recombinant genome (see Figure 10).

In the mating between VGII **a** (CBS10090) x VGIII α (NIH312), we isolated a total of 16 progeny. Mitochondrial amplification of the *ATP6* gene (mitochondrial) revealed that 100% of the progeny exclusively inherited the **a** mitochondrial genome (Figure 3B). Based on FACS analysis, we found that ∼half (7/16) were haploid (1N) and the rest (9/16) were diploid (2N) although this level of analysis cannot distinguish aneuploid isolates (Figure 3A). When each of the progeny was analyzed at eight unlinked MLST loci, including one sex-specific marker (*SXI1/SXI2*), 5/16 showed no signs of nuclear exchange (all α/haploid) but all five carried an **a** mitochondrial genome (Figure 3B). This indicates that these five mitochondrial exchange strains are the product of blastospores (i.e., monokaryotic yeast budding off of dikaryotic hyphae). We also show that two isolates harbor alleles from both nuclear genomes, have uniparental mitochondrial inheritance from the **a** parent, and also are haploid by FACS analysis, indicating that these two isolates were produced via meiosis, although one of the two shows increased levels of inheritance from the VGIII parent (Progeny 2 has 7/8 MLST loci from the VGIII parent while P3 has 4/8 MLST loci from the VGIII parent). The 9/16 remaining isolates show signs of aneuploidy: they all retain markers with sequences from both parental nuclear genomes and are all 2N or greater than 1N based on FACS analysis (Figure 3). These nine isolates also show self-fertility and 7/9 have both sex determining alleles (Figure 3B). The remaining two progeny (1 and 13) show no amplification of the *SXI2* **a** allele, however, these are self-fertile α isolates likely exhibiting robust α-α unisexual reproduction. In all of the isolates, the *CAP10* locus specifically amplified the VGIII allele, again suggesting either an amplification bias or the loss of this region of chromosome 11.

### Molecular Characterisation of Progeny from Additional Outgroup Crosses

**Figure 4:**
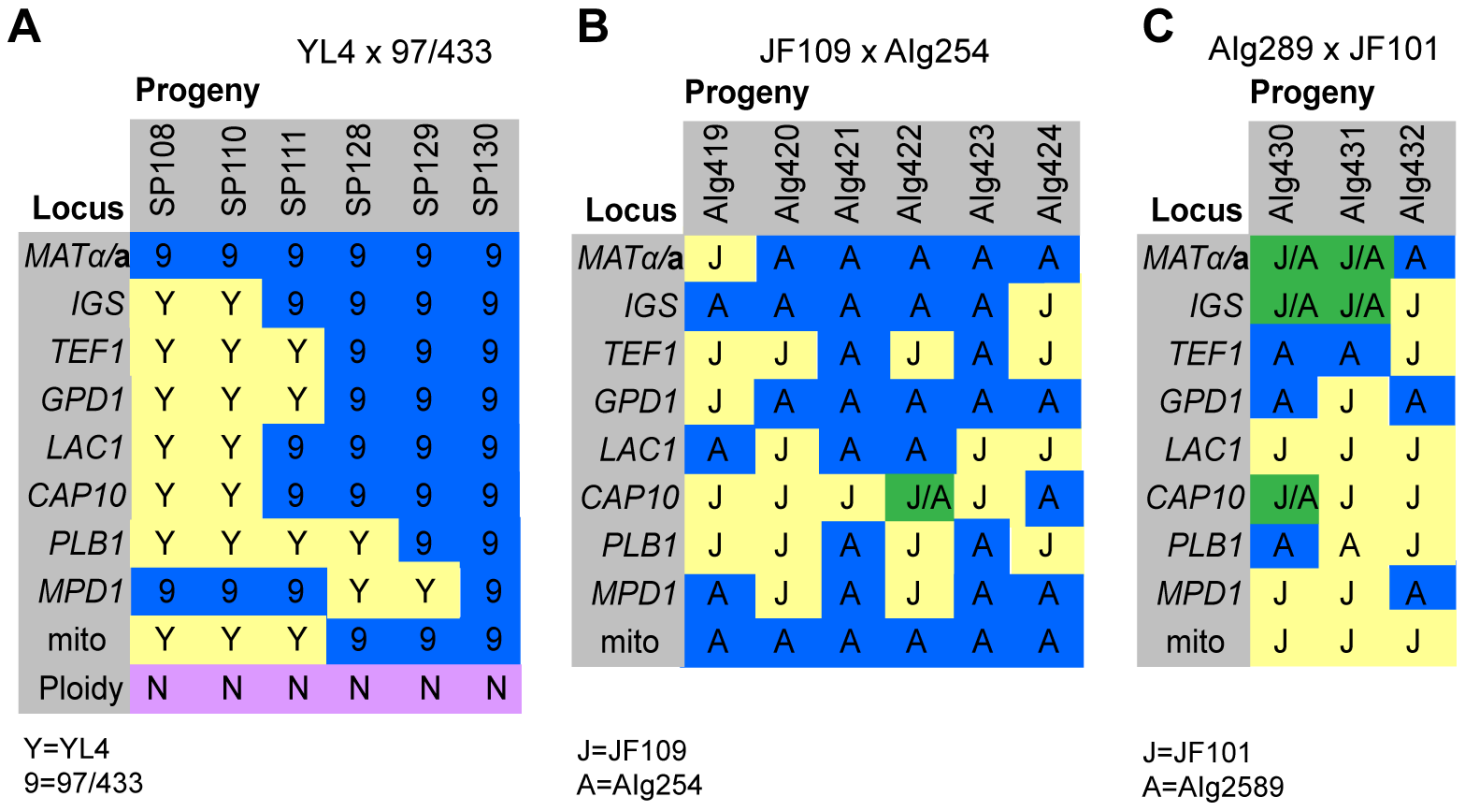
Molecular characterisation of progeny from marked outgroup crosses. **A**) Allele distribution in progeny of crosses YL4 x 97/433, **B**) AIg289 x JF101 and **C**) AIg254 x JF109. Blue 9 = allele from 97/433; Blue A = allele from AIg289 or AIg254; yellow Y = allele from YL4; yellow J = allele from JF101 or JF109,. One additional marker segregating in the AIg289 x JF101 cross is resistance to 5-fluorouracil. AIg420 is sensitive to this chemical; the other five progeny are resistant.

In the mating between VGIII α (97/433) x VGII **a** (YL4), we isolated a total of 7 progeny as described above. None of the progeny showed signs of self-fertility. Nuclear markers indicated that all progeny except SP130, which received all tested alleles from the VGIII α parent (97/433), were recombinant. Amplification of the *ATP6* gene (encoded by the mitochondrial genome) revealed that 4/7 of the progeny inherited the **a** and 3/7 the α mitochondrial genome (Figure 4A) indicating non-uniparental inheritance in this outgroup cross.

For the crosses with marked strains VGII α (AIg254) x VGIII **a** (JF109) and VGIII α (JF101) x VGII **a** (AIG289) three and six viable progeny were isolated. (Figure 4B&C). All nine progeny were recombinant compared to the parental isolates. They also all had inherited their mitochondrial genotype from the **a** parent.

Collectively, our molecular and phenotypic findings indicate that the rate of successful meiosis is low during VGII x VGIII mating with only 2/16 viable progeny being haploid recombinants in a VGII **a** x VGIII α mating and all of the viable progeny from the VGII α x VGIII **a** cross being diploid (2N). Thus, both sets of crosses indicate the presence of a restrictive barrier in meiosis due to cryptic speciation between molecular types VGII and VGIII. Compared to the high germination rate observed between VGIII x VGIII F1 progeny [32] both crosses produced few viable progeny. Although more than 163 spores that could be individually manipulated were produced in VGII x VGIII crosses, the spores did not germinate, suggesting that most progeny were largely inviable, as has been previously reported for sexual crosses between the related species *Saccharomyces cerevisiae* and *S. bayanus* [45], although at lower frequency.

### Inheritance of Macrophage Interaction Traits during Outgroup Crosses R265 x B4546

Given the involvement of mitochondria in cryptococcal hypervirulence [21] we considered whether mitochondrial genotype is the sole determinant of hypervirulence. If so, then we would anticipate the intracellular proliferation rate and mitochondrial tubularisation pattern of the progeny from these crosses to match that of the mitochondrial-donor parent.

**Figure 5:**
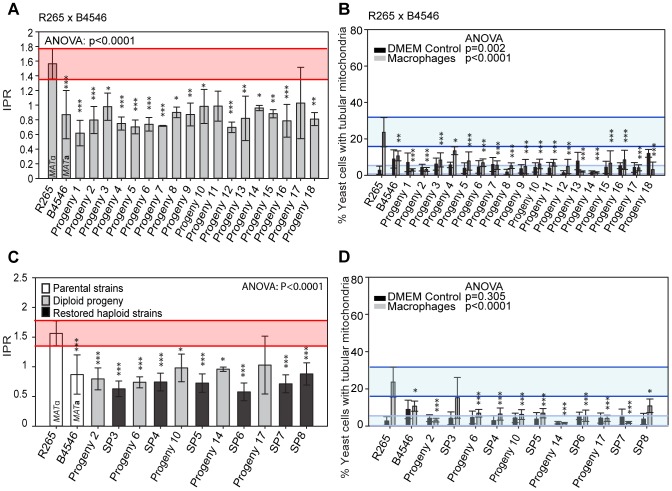
Macrophage interaction of progeny from outgroup crosses between R265 and B4546. (**A and B**) Diploid and (**C and D**) haploid progeny were tested for their ability to proliferate (**A and C**) and form tubular mitochondria within macrophages (**B and D**) and results compared to their respective parental strains. The **a** parent (B4546) shows a low intracellular proliferation rate (IPR) and a low percentage of tubular mitochondria while the α parent (R265) shows a high IPR and a high percentage of tubular mitochondria when growing within macrophages. All progeny show a low IPR and a low percentage of mitochondrial tubularisation. Shaded red area (**A and C**) indicates the standard error in the IPR of the hypervirulent parent. In (**C and D**), the light blue line indicates the level of tubularisation *in vitro* for the hypervirulent parent, and the dark blue line indicates the corresponding parental level of tubularisation within macrophages. Asterisks indicate significant differences between strains compared to the high IPR parental strain (R265) (* p<0.05, ** p≤0.01, *** p≤0.001).

Indeed in the outgroup cross R265 x B4546, all 18 hybrid diploid progeny showed intracellular proliferation rates similar to that of the low-virulence *MAT* **a** (mitochondrial donor) parent B4546 (Figure 5A). In addition, none of these F1 strains were able to trigger extensive mitochondrial tubularisation in response to engulfment by a host macrophage (Figure 5B), suggesting that the replacement of the R265-type mitochondrion with that from B4546 eliminated the hypervirulence trait in these progeny.

Because the progeny from these cross were all diploid, we tested whether this hybrid nuclear genotype may be ‘masking’ virulence phenotypes. However, when we “restored six” of the strains to haploidy via repeated rounds of mitotic passage, both mitochondria tubularisation and intracellular proliferation rates remained low (Figures 5C&D and Figure S2A).

### Inheritance of Macrophage Interaction Traits during Outgroup Cross CBS10090 x NIH312

**Figure 6:**
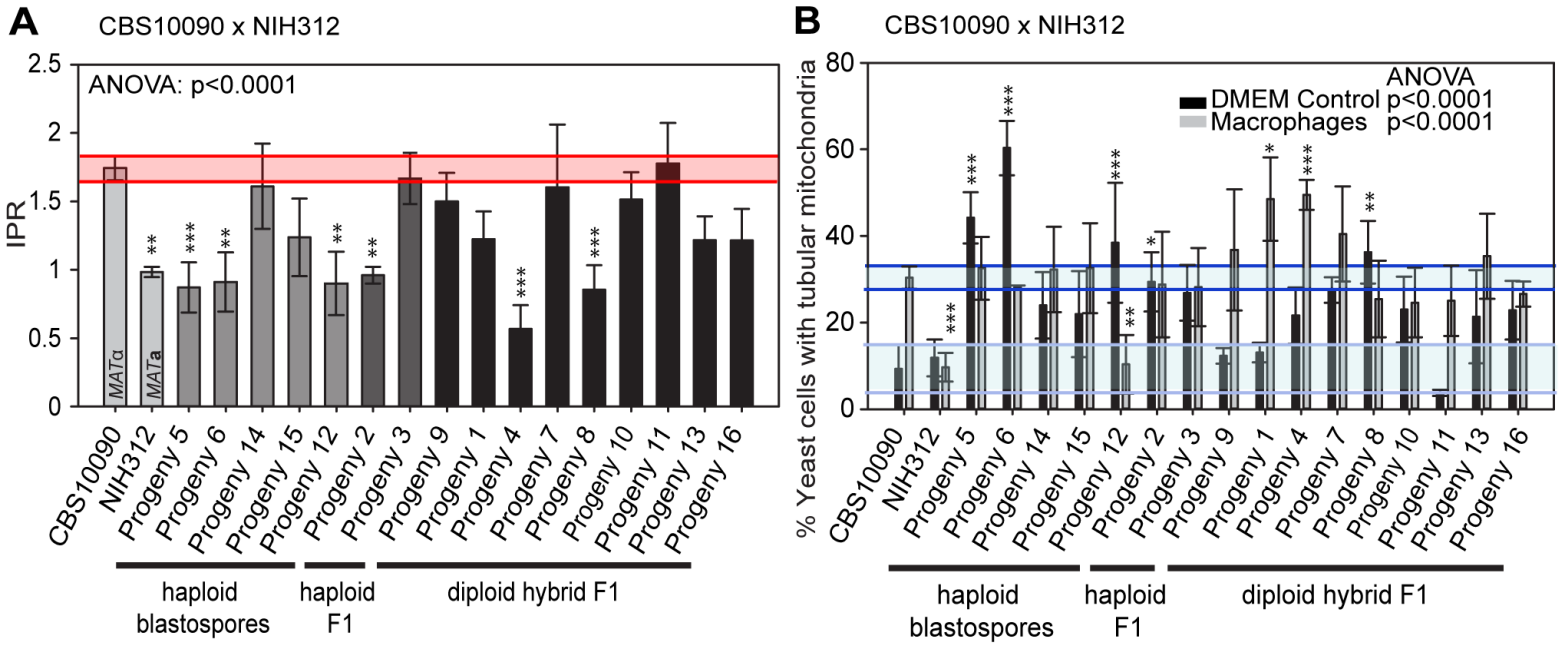
Macrophage interaction of progeny from outgroup crosses between CBS10090 and NIH312. Progeny were tested for their ability to **A**) proliferate and **B**) form tubular mitochondria within macrophages and the results were compared to their respective parental strains. The α parent (NIH312) shows a low IPR, while the **a** parent (CBS10090) shows a high IPR. In **A**), bars are shaded according to whether they are haploid blastospores (light grey), haploid recombinants (mid grey) or diploids (black) and the shaded red area indicates the standard error in the IPR of the hypervirulent parent. In **B**), the light blue line indicates the level of tubularisation *in vitro* for the hypervirulent parent, and the dark blue line indicates the corresponding parental level of tubularisation within macrophages. There is clear evidence for misregulated tubularisation in most of the progeny arising from this cross. Asterisks indicate significant differences between strains compared to the high IPR parental strain (R265) (* p<0.05, ** p≤0.01, *** p≤0.001).

In contrast to the R265 x B4546 cross, the cross between CBS10090 and NIH312, in which genotyping indicated all offspring carried mitochondria from the hypervirulent *MAT* **a** strain CBS10090, yielded F1 strains showing a wide range of intracellular proliferation rates (Figure 6A). Notably, two haploid recombinant offspring (Progeny 2 and 3) carry mitochondria from the virulent (CBS10090) parent, and yet only one (Progeny 3) shows a high intracellular proliferation rate. Thus a VGII mitochondrial genome, at least alone, is not sufficient to confer hypervirulence in this context.

Interestingly, progeny derived from haploid blastospores are isolates in which the nuclear genome is identical to the α parent NIH312, but the mitochondrial genome has been inherited from the **a** parent CBS10090. Such isolates show variable intracellular proliferation rates and tubularisation behaviour, e.g. Progeny 14 presents with IPR similar to the hypervirulent **a** parent CBS10090, whereas Progeny 5, 6, 15, and 12 proliferate less well within macrophages. This indicates that additional mechanisms might contribute to hypervirulence. In particular the sex induced silencing pathway becomes activated in blastospore progeny produced during the sexual cycle [46]. Thus, epigenetic processes might also contribute to altering biological properties of blastospore progeny, in addition to the exchange of the mitochondrial genome, leading to modified virulence phenotypes in blastospore progeny that are identical in their nuclear and mitochondrial genomes, yet differ phenotypically.

Lastly, we note that recombinant progeny from this cross no longer showed concordance between intracellular proliferation and mitochondrial tubularisation rates upon engulfment, with many strains showing high levels of tubularisation even under control conditions (Figure 6B and Figure S2B).

### Inheritance of Macrophage Interaction Traits in Additional Outgroup Crosses

**Figure 7:**
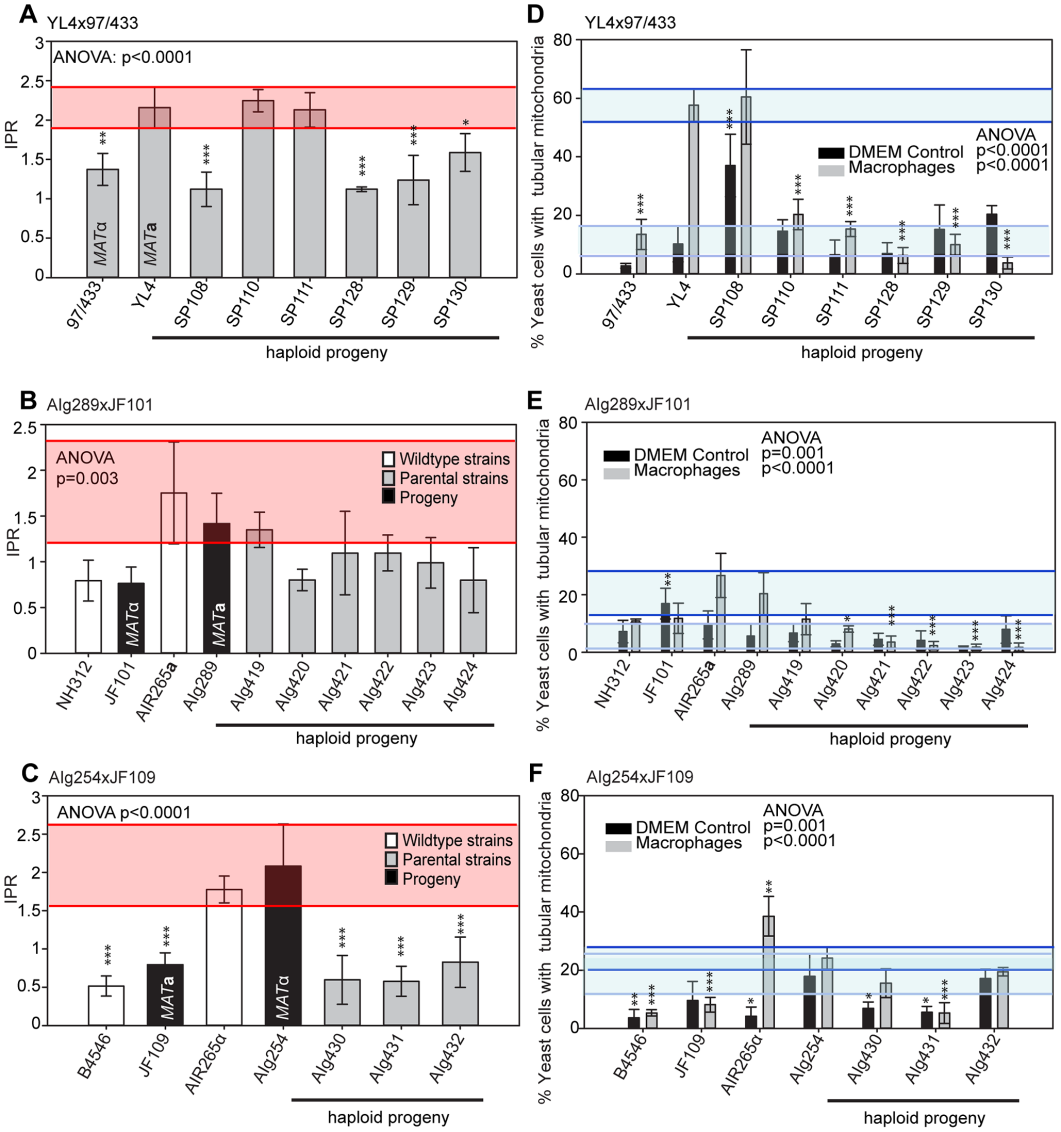
Macrophage interaction of progeny from marked outgroup crosses YL4 x 97/433, AIg189 x JF101 and AIg254 x JF109. Progeny were tested for their ability to proliferate (**A–C**) and form tubular mitochondria within macrophages (**D–F**) and the results were compared to their respective parental strains. **A–C**) The shaded red area indicates the standard error in the IPR of the hypervirulent parent. In (**D–F**), the light blue line indicates the level of tubularisation *in vitro* for the hypervirulent parent, and the dark blue line indicates the corresponding parental level of tubularisation within macrophages. Asterisks indicate significant differences between strains compared to the high IPR parental strain (R265) (* p<0.05, ** p≤0.01, *** p≤0.001).

To independently verify these observations, we undertook additional crosses using marked strains, as described above. Crosses between a hypervirulent *MAT* **a** and a low virulence *MAT*α parent resulted in range of intracellular proliferation rates (Figure 7A&B) whereas the reverse cross between a hypervirulent *MAT*α and a low virulence *MAT* **a** parent only produced progeny with low intracellular proliferative capacity (Figure 7C). Within these outgroup crosses, we also observed misregulated mitochondria (Figure 7D–F and Figure S2C–E).

Taken together, the data from these outgroup crosses thus strongly suggest that:

- The inheritance of mitochondria from a hypervirulent parent is, alone, not sufficient to confer high intracellular proliferation rates and,
- Multiple nuclear genomic regions are likely to interact with the mitochondrial genome to regulate hypervirulence in this group.

### Molecular Characterisation of Progeny from Ingroup Crosses

**Figure 8:**
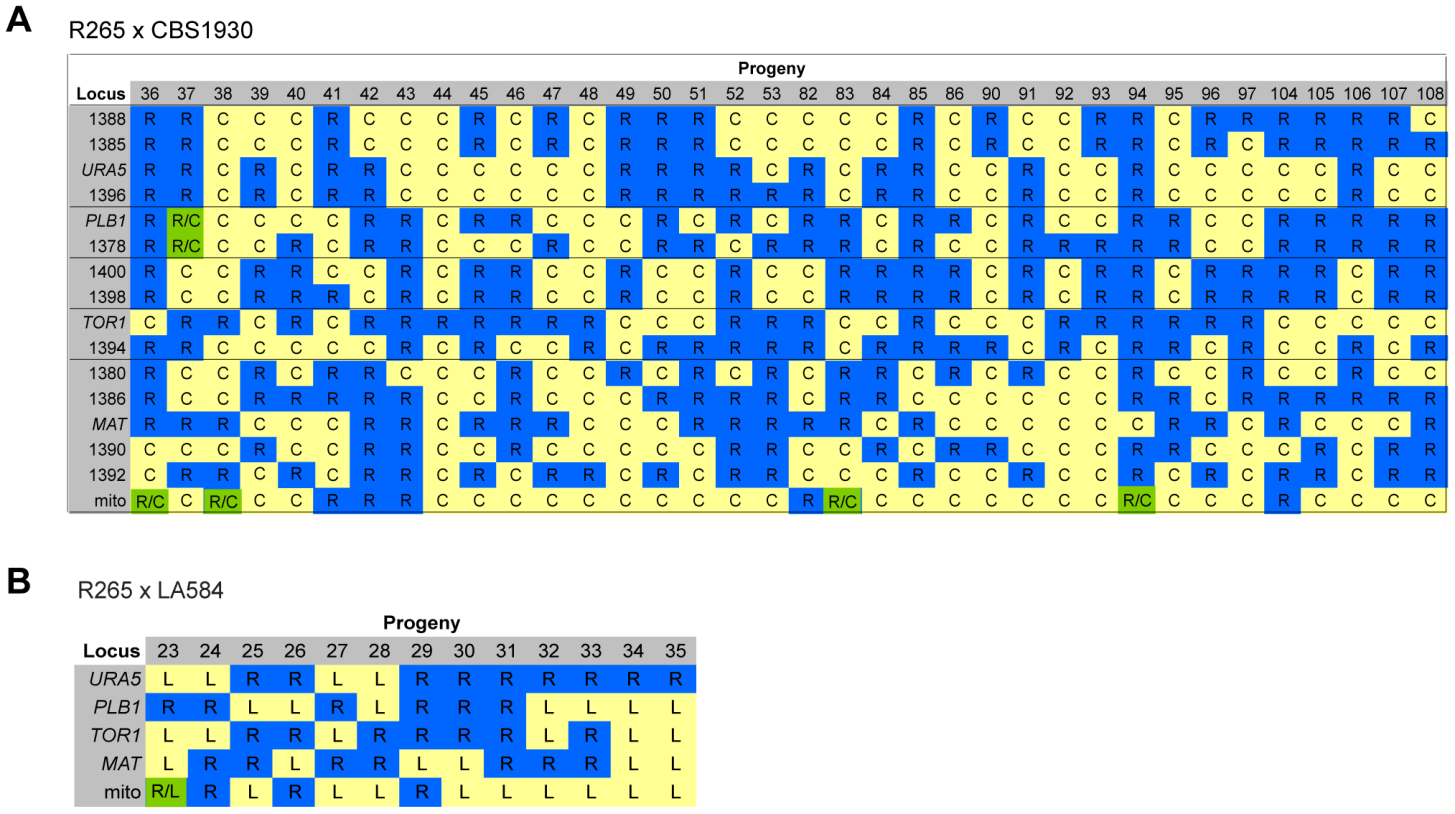
Molecular characterisation of progeny from ingroup crosses. **A**) Allele distribution in progeny of crosses R265 x CBS1930 and **B**) R265 x LA584. Blue R = allele from R265; yellow C = allele from CBS1930; yellow L = allele from LA584; green R/C = heterozygous for both alleles. Lines delineate sets of markers located on the same chromosome. Loci are provided as gene names or as the four digits of one primer used for amplification (ALID####, Table 3).

For the two ingroup VGII x VGII crosses (CBS1930 x R265 and LA584 x R265), nuclear markers indicated that all progeny were recombinant, although one (#37) from the cross between CBS1930 and R265 is likely aneuploid, because for one chromosome both parental alleles were amplified. Remarkably, however, for both crosses significant numbers of progeny inherited their mitochondria from the unexpected (R265, *MAT*α) parent: 9/36 for the cross with CBS1930 (Figure 8A) and 4/13 for the cross with LA584 (Figure 8B). Thus, it appears that, in contrast to outgroup crosses, crosses within the VGII clade produce a high proportion of viable recombinant progeny but that non-uniparental inheritance of mitochondria (i.e. in which either parent can donate mitochondria to daughter cells, but not at the same time) occurs more frequently than anticipated (25–30% compared to 5% in previous studies). This is analogous to the situation that occurs in atypical diploid-haploid crosses, in which mitochondria are inherited from the *MAT*α parent at a high rate [47].

### Inheritance of Macrophage Interaction Traits following Ingroup Crosses

**Figure 9:**
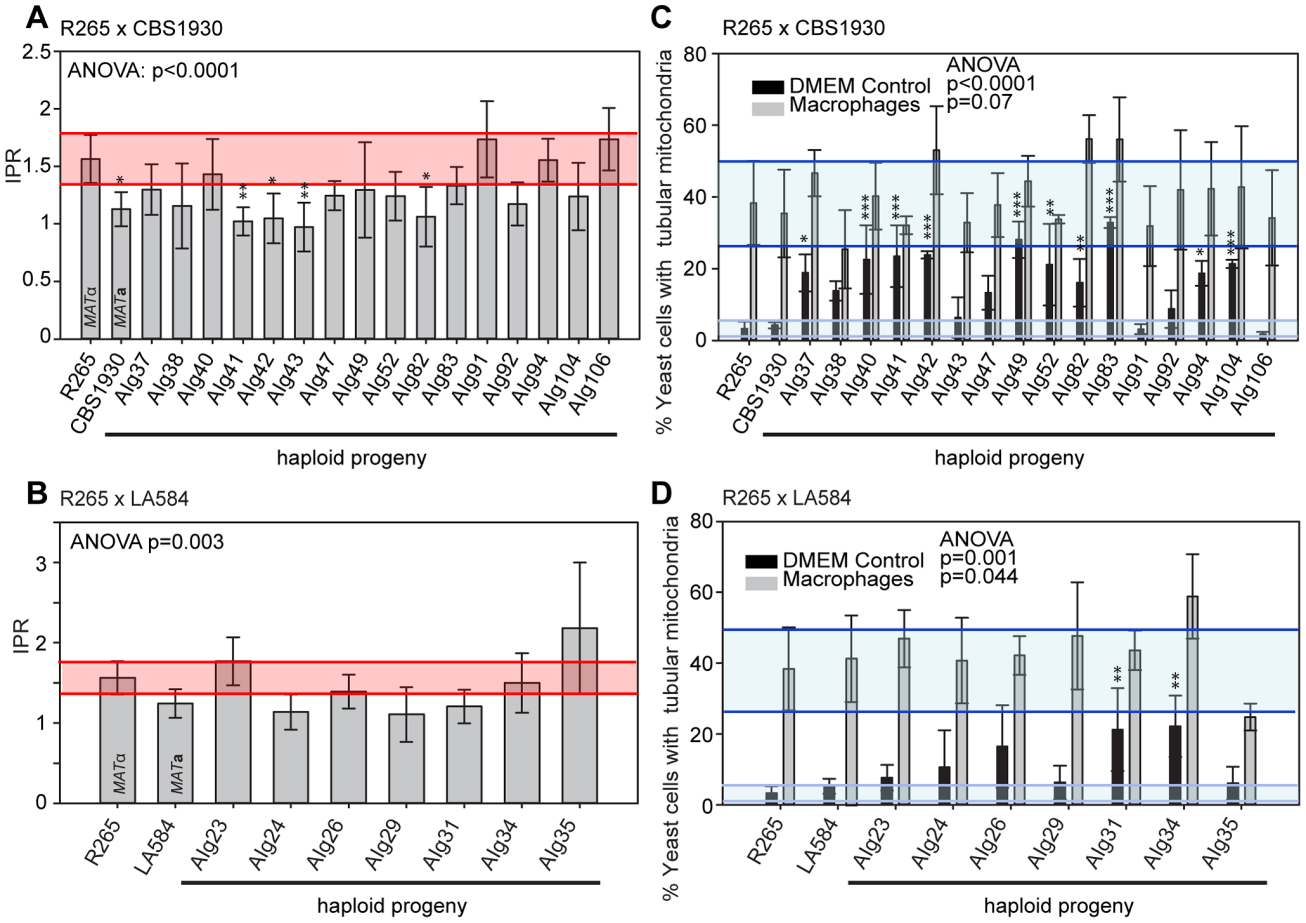
Macrophage interaction of progeny from ingroup crosses CBS1930 x R265 and LA584 x R265. Progeny were tested for their ability to proliferate (**A and B**) and form tubular mitochondria within macrophages (**C and D**) and the results were compared to their respective parental strains. (**A and B**) The shaded red area indicates the standard error in the IPR of the hypervirulent parent. In (**C and D**), the light blue line indicates the level of tubularisation *in vitro* for the hypervirulent parent, and the dark blue line indicates the corresponding parental level of tubularisation within macrophages. Asterisks indicate significant differences between strains compared to the high IPR parental strain (R265) (* p<0.05, ** p≤0.01, *** p≤0.001).

In contrast to the situation with outgroup crosses, several ingroup crosses resulted in a significant number of progeny that exhibited intracellular proliferation rates that were as high or even higher than the hypervirulent *MAT*α parent (R265), despite inheriting their mitochondrion from a lower-virulence *MAT* **a** parent (Figures 9A&B). Conversely, several progeny inherited the mitochondria from the hypervirulent α parent R265 and yet displayed low intracellular proliferation rates. Thus, hypervirulent phenotypes can spread within VGII independently of the mitochondrial genotype. We had anticipated that in these crosses the progeny would inherit mitochondria from a lower virulence *MAT* **a** parent (CBS1930 or LA584) but, as described above, in fact both crosses demonstrated a higher rate of mitochondrial inheritance from the *MAT*α parent (25 to 30%), indicating that mitochondrial inheritance is not strictly uniparental in this group.

Interestingly, unlike the CBS10090 x NIH312 outgroup cross (Figure 7), both ingroup crosses produced progeny that remained able to correctly tubularise their mitochondria in response to phagocyte engulfment (Figures 9C&D and Figure S2F&G), which most likely explains the ability of these recombinant progeny to continue to proliferate rapidly within host cells.

Increased mitochondrial genome copy number or a higher number of mitochondria can affect mitochondrial inheritance. Similarly, the larger cell size of one parent might lead to an increased cytoplasmic and/or mitochondrial contribution to progeny. For instance, hyper-suppressive *RHO* mutants of *S. cerevisiae* exhibit deletions in the mitochondrial genome and are ‘petite’ variants. However, the *S. cerevisiae* mitochondrial genome replicates faster than the wild type and consequently, when crossed with wild type, all progeny inherit the mutant mitochondrion [48]–[50]. To test whether such a phenomenon may account for the non-uniparental mitochondrial inheritance we observed during in-group crosses, we measured the size of our parental strains under control conditions *in vitro* and after macrophage passage. However, cell size does not appear to be a contributing factor for changes in virulence (data not shown). In addition, previously published data on mitochondrial DNA copy number [21] showed no increase of mitochondrial genetic information and hence makes it unlikely that a higher copy number from R265 leads to a ‘leak’ from the α parent in those crosses.

### Recombination of Mitochondrial Genomes in *C. gattii*

**Figure 10:**
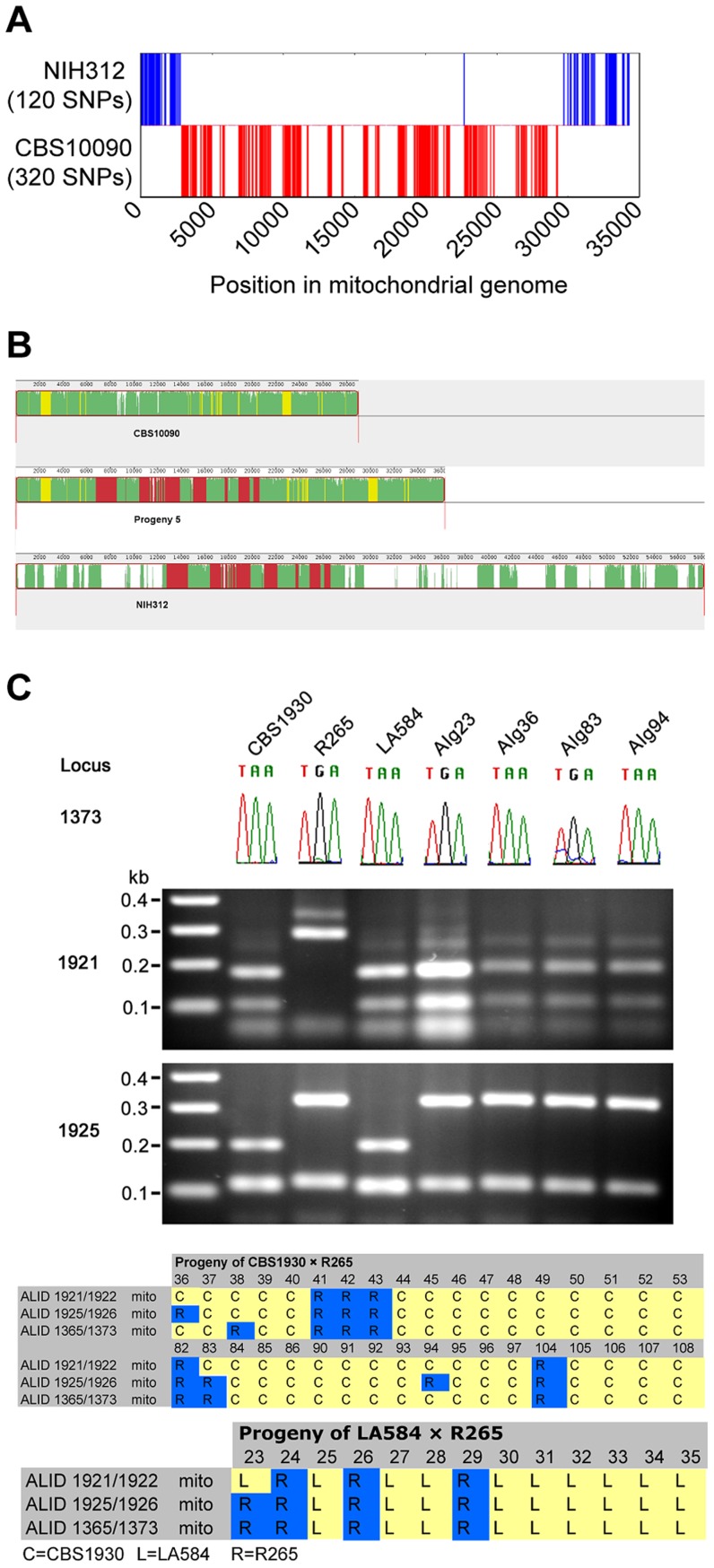
Mitochondrial recombination occurs in *C. gattii* crosses. **A**) Progeny 5 from cross CBS10090 (VGII) x NIH312 (VGIII) has a mixture of SNPs distributed from both strains. **B**) Mitochondrial genome sequences of CBS10090 (top), Progeny 5 (middle) and NIH312 (bottom) were aligned with Progressive Mauve [89]. Syntenic regions are marked in different colors. Green: conserved in all genomes, yellow: conserved in CBS10090 and Progeny 5 only, red: conserved in NIH312 and Progeny 5 only, white: unique to each genome. **C**) Four progeny from VGII crosses (AIg23 from R265 x LA584; AIg36, AIg84 and AIg94 from R265 x CBS1930) have mixtures of alleles from each parent. Locus 1373 was amplified and sequenced, with the chromatogram used to illustrate the A–G polymorphism. Locus 1921 and 1925 were amplified by PCR, digested with restriction enzymes, and the products resolved on agarose gels.

The surprising result of biparental mitochondrial inheritance in the ingroup crosses (mitochondria inherited from **a** parent 70–75% of the time and from the α parent 25–30% of the time), in contrast to uniparental inheritance from only the **a** parent in outgroup crosses, prompted us to further investigate the unexpected phenotypes of progeny from the outgroup cross between CBS10090 and NIH312. By utilising whole-genome sequence data for the two parental strains and Progeny 5, which had been made available as part of a larger sequencing project, we found 440 mitochondrial single nucleotide polymorphisms (SNPs) that differed between CBS10090 and NIH312. Progeny 5 shared 320 SNPs with CBS10090 and 120 with NIH312. Aligning these sites across the 34 kb mitochondrial genome showed that, with the exception of a single SNP, all sites from a single parent formed contiguous blocks (Figure 10A). Thus this pattern of polymorphisms represents very strong evidence for mitochondrial recombination in Progeny 5 from the VGII x VGIII outgroup cross CBS10090 x NIH312 (Figure 10B). In addition, by using three mitochondrial markers to assess the inheritance of mitochondrial DNA in the VGII x VGII crosses, we identified four examples of recombinant mitochondrial genotypes (Figure 10C). These findings support previous evidence for mitochondrial recombination both in *C. neoformans* [27], [51] and *C. gattii* [52].

## Discussion

In this study we conducted a systematic analysis to test the potential spread of hypervirulence in *C. gattii*. In crosses between a hypervirulent VGII strain and strains from a different clade, VGIII, it appears that a mitochondrial genotype originating from within the outbreak is necessary, but not sufficient, to confer hypervirulence. Thus simple transmission of a mitochondrial lineage is unlikely to spread outbreak traits to a new population of *C. gattii*.

We generated multiple sets of VGII x VGIII F1 progeny, one from a VGII **a** x VGIII α mating, and the other from a VGII α x VGIII **a** mating. The first conclusion from the progeny sets was that germination was very infrequent (less than 1%, 0/163 spores), supporting the hypothesis that these molecular types are cryptic species manifesting strong reproductive barriers. To circumvent this, isolation of progeny had to be done without individual spore dissections (selecting probable progeny from spore-dense regions); resulting in a need to clearly type all progeny using a multilocus sequence typing (MLST) or PCR-RFLP based approaches.

Several independent studies have shown that, based on sequence analysis, there is no allelic exchange observed between the four molecular types of *C. gattii*, leading to a hypothesis that they are independent species within the pathogenic *Cryptococcus* species complex and adhere to a phylogenetic species concept [11], [24], [33], [34], [53]. The results presented in this manuscript demonstrate that there are clear reproductive barriers between isolates from two of the molecular types examined, VGII and VGIII. This reproductive barrier is post-zygotic, and supported with examples from both VGII α x VGIII **a** as well as VGII **a** x VGIII α crosses. Therefore, these two lineages appear to also adhere to a biological species concept. Specifically, analysis of progeny from such crosses showed predominantly hybrid diploids, followed by mitochondrial exchange strains (i.e., blastospores), and finally only 1–2/34 (3–6%) haploid recombinants. This post-zygotic barrier parallels studies examining progeny isolated from sexual crosses between the related species *Saccharomyces cerevisiae* and *S. bayanus*, whereby viable spores from tetrads were found at a frequency of about 1/10,000 [45]. Furthermore, similar post-zygotic barriers between closely related species from within both the *Microbotryum* and *Neurospora* genera and AD hybrids in *C. neoformans* result in low levels of viable progeny [54]–[56].

The finding that a majority of progeny are diploid hybrids is intriguing, as it suggests that, both environmentally and clinically, there may be a potential for inter-molecular type hybrids, such as VGII x VGIII hybrids, to occur. This would parallel several seminal studies examining both *C. neoformans* var. *neoformans/C. neoformans* var. *grubii* (AD) and *C. neoformans/C. gattii* (BD and AB) hybrids [41], [54], [57]–[62]. These studies show that the hybrids are able to infect hosts, as several are clinical in origin, and our IPR assays, at a minimum, suggest that VGIII x VGII diploid hybrids can be virulent.

Mitochondrial inheritance in *C. neoformans*, as in most eukaryotes, is uniparental [63], [64], with progeny inheriting mitochondria from the *MAT* **a** parent [29]. However, environmental factors such as high temperature and UV irradiation can lead to biparental inheritance and recombination of mitochondrial DNA [65]. Our data indicate that, at least within VGII, inheritance of mitochondria from either parent (rather than only one) can occur relatively frequently (25–30%), even in the absence of such stresses, and that recombination can occur for the mitochondrial genome. Previous studies have shown that while **a**-α mating leads to uniparental inheritance, regulated by the mating type-specific homeodomain genes *SXI1*α and *SXI2* **a**, α-α mating has biparental inheritance, which enables increased mitochondrial genome recombination [66]. Other recent studies have demonstrated roles for genes not within the mating type locus in the control of uniparental inheritance [67]. Furthermore, in a congenic VGII *MAT* **a**/*MAT*α strain pair in the R265 background, uniparental inheritance was observed [68]. This supports an interesting hypothesis whereby α-α mating may contribute to the formation of recombinant mitochondrial genomes with higher predispositions for virulence, possibly explaining the hypervirulence among specific VGII genotypes.

In addition to demonstrating biparental organelle inheritance in *C. gattii*, we provide clear genomic evidence for mitochondrial recombination following mating, in support of previous MLST/AFLP data that suggested the occurrence of mitochondrial recombination at the population level [38], [52]. Together, these data suggest that there may be less stringent control over uniparental mitochondrial inheritance by the mating type locus in the *C. gattii* VGII lineage as compared to much stricter uniparental mitochondrial inheritance in *C. neoformans*.

Whilst the overall populations of strains still showed a significant correlation between intracellular proliferation and mitochondrial tubularisation (Figure S2E), one striking observation from these crosses was the high frequency of ‘misregulated’ mitochondria, in which mitochondrial tubularisation (which, in outbreak strains, is a response to the stressful environment of the host cell involving mitochondrial fusion) occurs even under control growth conditions (Figure 5). Mitochondrial morphology is regulated by proteins encoded within the nuclear genome and, in *S. cerevisiae*, fusion events are controlled by a protein complex consisting of Fzo1p, Mgm1p, and Ugo1p [69]–[72]. Thus effective interaction of this machinery might be interrupted by non-compatible mitochondrial and nuclear genomes, reducing the ability of recombinant progeny to regulate virulence traits.

In contrast, for crosses between parents within the VGII clade, hypervirulence traits appear to spread easily and are no longer strictly dependent on the presence of a mitochondrial genotype originating from within the outbreak. Thus many, perhaps all, VGII mitochondria are capable of tubularising under host conditions and driving rapid intracellular proliferation, and therefore virulence. This presumably reflects the compatibility of nuclear and mitochondrial ‘cross-talk’ across VGII genotypes, allowing mitochondrial morphology to be correctly regulated in recombinant progeny. This may explain why multiple, distinct outbreaks of disease have all been caused by VGII isolates that differ in their genotype [11].

Our experimental model indicates that hypervirulence in *C. gattii* is a complex, multigenic trait, requiring regions of the mitochondrial genome and regions of the nuclear genome to confer hypervirulence, which can be attained by a variety of genetic combinations after sexual mating. Thus, there are potentially multiple routes by which such traits could disperse through the *C. gattii* population, suggesting that surveillance efforts should consider the possibility of independent outbreaks caused by distinct lineages of this pathogen. Our studies also provide phenotypic and molecular evidence that the VGII and VGIII molecular types of *C. gattii* are distinct species, separated by post-zygotic reproductive barriers. Finally, we provide evidence that mitochondrial inheritance in this species is more complex than currently appreciated, with both biparental mitochondrial inheritance and mitochondrial recombination being observed.

## Materials and Methods

### Yeast Strains, Mammalian Cells and Growth Conditions

*Cryptococcus gattii* strains (Table 1 and Table 2) used in this study were cultured in liquid or agar YPD media (1% peptone, 1% yeast extract, 2% D-(+)-glucose) for 24 h at 25°C rotating at 20 rpm prior to experiments [73]. Mammalian J774 cells were grown as described previously [73].

**Table 1:** Parental *Cryptococcus gattii* strains used in this study used for crosses.

Parental Strains	Mating Type	Molecular Type	Source	Additional Information	Reference
R265	alpha	VGIIa	Human	Clinical isolate (bronchial wash) from Duncan, British Columbia, Canada	[9]
CBS1930	a	VGII	Animal	Sick goat, Aruba	[90]
CBS10090	alpha	VGII	Human	Clinical isolate from immunocompromised patient, Greece	
NIH312	a	VGIII	Human	Clinical isolate, USA	
B4546	a	VGIII	Human	Clinical isolate from spinal fluid of patient with cryptococcal meningitis, USA	[90]
LA584	a	VGII	Human	Clinical isolate, CSF, Colombia	[91]
YL4	a	VGII		Possible progeny of 96/1120 x La55	[11]
97/433	alpha	VGIII		Clinical isolate from 17 year old female AIDS patient	[54]
JF101	alpha	VGIII		*crg1::NEO* in NIH312 background	[32]
AIR265a	a	VGII		Wildtype congenic pair	[68]
AIg289	a	VGII		*bwc2::NAT fur1* in R265 background	[68]
JF109	a	VGIII		*crg1::NEO* in B4546 background	[32]
AIR265α	alpha	VGII		Wildtype congenic pair	[68]
AIg254	alpha	VGII		*bwc2::NAT* in R265 background	[68]

**Table 2:** Crosses conducted for the study and resulting progeny.

Crosses	Progeny
R265 x B4546	Progeny 1–18
	SP3-SP8
CBS10090 x NIH312	Progeny 1–16
R265 x LA584	AIg23
	AIg24
	AIg26
	AIg29
	AIg31
	AIg34
	AIg35
R265 x CBS1930	AIg37
	AIg38
	AIg40
	AIg41
	AIg42
	AIg43
	AIg47
	AIg49
	AIg52
	AIg82
	AIg83
	AIg91
	AIg92
	AIg104
	AIg106
YL4 x 97/433	SP108
	SP110
	SP111
	SP128
	SP129
	SP130
AIg289 x JF101	AIg419-424
AIg254 x JF109	AIg430-432

### Crosses

For outgroup crosses between VGII **a** CBS10090, VGII α R265, VGIII **a** B4546, and VGIII α NIH312, mating assays were conducted on V8 media (5% V8 juice, 0.5 g/L KH_2_PO_4_, 4% agar; pH = 5) to generate spores for progeny analyses. Isolates were incubated at room temperature in the dark for 2–4 weeks in dry conditions. Fertility was assessed by light microscopy to identify basidiospore formation at the periphery and surface of the mating patch. To collect progeny from the crosses, basidiospores were isolated with a micromanipulator as described previously with the slight modification that due to low germination rate, other suspected resultant colonies were collected from areas where groups of basidiospores were collected [74], [75]. To summarize, spore-dense regions were collected using a glass Pasteur pipette and spread on a YPD agar plate. In total, the progeny set from the CBS10090 x NIH312 cross yielded 16 collected progeny and the R265 x B4546 cross yielded 18 collected progeny. Progeny from crosses YL4x97/433, AIg289xJF101 and AIg254xJF109 were established as described above. For AIg289 x JF101 and AIg254 x JF109, the cell mixture was plated onto YPD supplemented with nourseothricin and G-418 (both at 100 µg/ml).

To derive haploid progeny (SP3-SP8), the diploid progeny were passaged on YPD agar every 48 hours for 24 days (total 12 passages). A single colony from the previous passage was used to initiate the next passage. FACS analysis was performed on five colonies from every passage to determine the ploidy. Serial passaging was stopped when cells from at least one of the five colonies were found to be haploid. Cells from these haploid colonies were used to grow overnight cultures in liquid YPD, which were stored frozen at −80°C in glycerol and subsequently used for MLST and virulence analyses.

Crosses between R265 and CBS1930 or LA584 were established on V8 juice agar medium (pH unadjusted). Yeasts were mixed on the plate and examined 2–4 weeks later for the presence of basidia and basidiospore chains. Spores and surrounding parental yeasts were transferred using a gel-loading tip to YPD agar medium. Individual basidiospores were micromanipulated with a dissecting microscope. Genomic DNA from progeny was prepared by disrupting cells in buffer (10 mM Tris-HCl [pH 7.5], 10 mM EDTA, 0.5 M NaCl, 1% SDS)+½ volume chloroform with 425–600 µm glass beads, aided by two rounds of vortexing and freezing at −20°C. After centrifugation, the DNA in the supernatant was precipitated with an equal volume of isopropanol. For preparing larger quantities of DNA, a CTAB-based extraction buffer was used on lyophilized cells harvested from 50 ml cultures [76].

### Identification and Detection of Polymorphic Regions between Strains

For the outgroup crosses sequence data for the MLST alleles of the parental isolates were previously published (Table S1) [11], [24]. For each F1 progeny isolate, DNA was isolated (Epicentre), and genomic regions were PCR amplified (Table S2), purified (ExoSAP-IT, Qiagen), and sequenced. Sequences from both forward and reverse strands were assembled and manually edited using Sequencher version 4.8 (Gene Codes Corporation). Based on the sequences of the parental strains, alleles of the progeny were assigned to three distinct categories: exclusively from the α parent, exclusively from the **a** parent, or heterozygous. Heterozygosity at each allele was based on alignments with parental alleles and clear observations of multiple positive nucleotide traces in regions that differ between the two parental sequences, which are indicative of two unique sequences (i.e., one from each parent) being analyzed. Additionally, mitochondrial inheritance was assayed using *ATP6* mitochondrial specific forward (ACTTGCGGCTGAATGATAAAATCTAA) and reverse (GTGGAGATGTAATAAAGTGTGTCATG) primers, whereby the product (including the 5′ UTR and part of the ORF) from the VGIII (**a** and α) mitochondrial genome is larger than the product from the VGII (**a** and α) mitochondrial genome [5], [11].

For progeny from crosses R265 x CBS1930 and R265 x LA584, polymorphic regions were identified in multilocus sequence typing (MLST) data in GenBank and by sequencing fragments of the CBS1930 genome. MLST differences between the strains were used for three PCR-RFLP markers [24]. To identify other polymorphic regions, a small genomic DNA library was constructed from DNA of strain CBS1930. HindIII restriction fragments were cloned into the HindIII site of plasmid pBluescript. The ends of the inserts were sequenced, and the sequence compared by BLASTn to the R265 strain genome database at the Broad Institute [77]. Either single nucleotide polymorphisms (SNPs) affecting restriction enzymes sites or with multiple differences between the two strains were used for the design of oligonucleotides primers for PCR amplification of alleles from either parent. PCR reactions, digested with restriction enzyme where necessary, were resolved on 1× TAE agarose gels. Primer sequences and details about the polymorphisms are in Table 3. Sections of the mitochondrial genome were amplified and sequenced from R265, CBS1930, and LA584 to identify a polymorphism.

**Table 3:** Markers used for analysis of progeny from R265 x CBS1930 and R265 x LA584.

Chr.	Supercontig number and position(s)	Primer name	Sequence of primer (5′-3′)	Detection
I	18 at ∼22,245–44,4480 (*MAT*)	N/A	N/A; *MAT*a or *MAT*α	Phenotype
M	13 at 174,953 (*PLB1*)	JOHE14974	CTCTCATTGTTCGCCGCTACT	NdeI
		JOHE14975	GGAAGCCGAGGTCTGATTTGG	
F	4 at 972,428 (*TOR1*)	JOHE15471	TTCGGTACCATCCTGAGTTAT	MseI
		JOHE15472	TTAGCCAAGGTCTTCCCACTG	
G	5 at 966,811 (*URA5*)	ALID1356	GTACTTCCTGACCTCTCGC	BsrGI
		ALID1357	GAGCTCATAAGCCAGTAG	
M	13 at 319,262–319,703	ALID1376	AATTTTGGAAGAATCTATGG	CBS1930
		ALID1378	TCTTCAACCATTCAGATGTG	
		ALID1377	AATTTTGGAAGAATCTATTC	R265
		ALID1379	TCTTCAACCATTCAGATGTA	
N	14 at 520,464–520,467	ALID1380	TGGCTCTGCAAGGTCAAG	Both alleles
		ALID1381	CCATCATTAACACGCTCTGC	CBS1930
		ALID1382	CCATCATTAACACGCTATGA	R265
G	5 at 453,154–453,169	ALID1383	ACGGTCTCCATCTCGAAC	Both alleles
		ALID1384	CGCTTCCTGCGAGCCAGC	CBS1930
		ALID1385	TTGCTGCTAGCCAGCTTC	R265
C	8 at 674,370	ALID1386	ATTTACCGGAGAAGTTCGTC	SacII
		ALID1387	GGAGCAATGGAAACTCGGTC	
G	5 at 252,641	ALID1388	ATGGAGAGGTCAACAAGC	HpyCH4V
		ALID1389	TCTCAAAGCAAGTCGGTG	
C	10 at 239,575	ALID1390	CCACTAATCGACTGGTCAGC	BspHI
		ALID1391	CAATAACGCAATCATAGACC	
J	21 at 35,753	ALID1392	ATTCTGGTCGTGCGAGACGC	Sau3AI
		ALID1393	AGTCCGGGTCAAGAGTCACC	
F	4 at 189,077	ALID1394	AGCTGATATAGAAGCTCTCC	SphI
		ALID1395	TAAGCTAGACGATGTGAAGG	
G	5 at 1,102,776	ALID1396	TTTCCCGACTAATGTGATGG	NdeI
		ALID1397	AGTCATTAGCAGCCGAGCTG	
H	6 at 915,271	ALID1398	GGCTAACATCACTGTTGTAG	AluI
		ALID1399	ATAAGGGTGACCTGAAGCTG	
H	6 at 707,230	ALID1400	CGAGAGGAGTGTCGTCTTAC	MspI
		ALID1401	AAGAGCAGACTCGGGATCAG	
mt	29	ALID1365	AACTACGGATGGATGATTCG	Sequencing with ALID1373
		ALID1373	AGTGAAGTGAGAAGAATCGG	
mt	29	ALID1921	CTACTTCTAGCTATGGTAGG	MseI
		ALID1922	ACACTATCTCGCATGTGTAG	
mt	29	ALID1925	CAAGTATGCCCTCTCTGG	TaqI
		ALID1926	TTGCTTAAAGGAGTGGAC	

Chromosome (Chr.) refers to that defined for strain WM276. Supercontig and the position(s) refers to the R265 genome sequence. Polymorphisms were detected as a phenotype (*MAT* **a** or *MAT*α) by crossing, as PCR products specific for alleles from each parent, by digestion of PCR products with the restriction enzymes listed, or by sequencing a PCR product. JOHE primers are from [24].

Analysis of nuclear and mitochondrial markers was conducted to test if progeny were recombinant was conducted. The strains from the cross between R265 and CBS1930 were assessed for 16 genetic markers. One was the mating type phenotype and 14 were PCR markers that amplified polymorphic parts of the nuclear genome. The markers are located on eight of the 14 chromosomes of *C. gattii* [77]. For the mitochondrial genome, the *COX1* and *COB1* genes were amplified and sequenced. A single nucleotide polymorphism was identified in intron 2 of *COB1* (submitted as GenBank accessions JX486912 and JX486913). Subsequently, the CBS1930 strain was subject to Illumina genome sequencing, and the mitochondrial genome analyzed for other differences between this strain and R265. Two other regions were used to track the inheritance of the mitochondrial genome in the VGII x VGII progeny.

### Ploidy Determination by Fluorescence Flow Cytometry

Cells were processed for flow cytometry as described previously, with slight modifications [54], [78]. Briefly, cells were harvested from YPD medium, washed once in phosphate-buffered saline (PBS) buffer, and fixed in 1 ml of 70% ethanol overnight at 4°C. Fixed cells were washed once with 1 ml of NS buffer (10 mM Tris-HCl (pH 7.5), 250 mM sucrose, 1 mM EDTA (pH 8.0), 1 mM MgCl_2_, 0.1 mM CaCl_2_, 0.1 mM ZnCl_2_) and then stained with propidium iodide (12.5 mg/ml) in 0.2 ml of NS buffer containing RNaseA (1 mg/ml) at 4°C for 16 h. Next, 0.5 ml of stained cells were diluted into 0.5 ml of 50 mM Tris-HCl (pH 8.0). Flow cytometry was performed on 10,000 cells and analyzed on the FL1 channel with a Becton-Dickinson FACScan (Duke University Medical Center Flow Cytometry Core Facility).

### Self-Filamentation Assays

Filamentation assays were conducted on V8 media (pH = 5) and filamentation agar [79]. Isolates were incubated at room temperature in the dark for 2–4 weeks in dry conditions. Filamentation was assessed by light microscopic examination for hyphae formation at the periphery and surface of the incubated patches. All assays were conducted on both media types. If there were no signs of filamentation after a four-week period, isolates were scored as having no self-filamentation phenotype.

### Cryptococcal Intracellular Proliferation Assay and Mitochondrial Staining

Macrophages were infected with yeast cells and intracellular proliferation monitored as previously described [80]. Cryptococcal mitochondrial morphology was determined as described previously [11]. In brief, to determine the intracellular proliferation rate (IPR) of individual strains following phagocytosis, J774 macrophage cells were exposed to cryptococcal cells that were opsonized with 18B7 antibody (a kind gift from Arturo Casadevall) for 2 hr as described previously [73]. Each well was washed with PBS in quadruplicate to remove as many extracellular yeast cells as possible and 1 ml of fresh serum-free DMEM was then added. For time point T = 0, the 1 ml of DMEM was discarded and 200 µl of sterile dH_2_O was added into wells to lyse macrophage cells. After 30 minutes, the intracellular yeast were released and collected. Another 200 µl dH_2_O was added to each well to collect the remaining yeast cells. The intracellular yeast were then counted with a haemocytometer. For the subsequent five time points (T = 18 hrs, T = 24 hrs, T = 48 hrs, T = 72 hrs), intracellular cryptococcal cells were collected and counted. For each strain tested, the time course was repeated at least three independent times, using different batches of macrophages. The IPR value was calculated by dividing the maximum intracellular yeast number by the initial intracellular yeast number at T = 0. We confirmed that Trypan Blue stains 100% of the cryptococcal cells in a heat-killed culture, but only approximately 5% of cells from a standard overnight culture. The mitochondrial morphology assays were conducted in a similar way to those in previous studies, with modifications [21]. *C. gattii* cells were grown overnight at 37°C in DMEM untreated or isolated from macrophages 24 hr after infection. The cells were harvested, washed with PBS twice and re-suspended in PBS containing the Mito-Tracker Red CMXRos (Invitrogen) at a final concentration of 20 nM. Cells were incubated for 15 min at 37°C. After staining, cells were washed three times and re-suspended in PBS. For each condition, more than 100 yeast cells per replicate for each of the tested strains were chosen randomly and analysed. To quantify different mitochondrial morphologies, images were collected using a Zeiss Axiovert 135 TV microscope with a 100× oil immersion Plan-Neofluar objective or a Nikon Eclipse T*i* Plan Apo VC 60× oil immersion objective. Both fluorescence images and phase contrast images were collected simultaneously. Images were captured with identical settings on a QIcam Fast 1394 camera using the QCapture Pro51 version 5.1.1 software. All images were processed identically in ImageJ and mitochondrial morphologies were analysed and counted blindly [11]. IPR and tubularisation data were analysed for statistically significant differences using one-way ANOVA analysis with multiple comparisons by Tukey Honestly Significant Difference (HSD) posthoc test. A p-value of <0.05 after controlling for multiplicity was considered to be statistically significant.

### Illumina Sequencing

Genomic DNA from *C. gattii* strains NIH312, CBS10090, and progeny 5 from the cross between NIH312 and CBS10090 was isolated with the EpiCentre MasterPure Yeast DNA Purification Kit according to a modified version of the instruction manual. Briefly, the strains were grown in liquid YPD media for 24 h at 25°C rotating at 20 rpm. Cells from 3 ml of culture were harvested by centrifugation at 17,000× g for 5 minutes. Cells were lysed in 300 µl of Yeast Cell Lysis solution by mechanical disruption with 0.1 mm silica spheres (FastPrep Lysing Matrix, MP Biomedicals) twice for 30 seconds at 6,800 rpm in a Precellys24 and incubation at 65°C for 15 minutes. Samples were cooled down on ice for 5 minutes and proteins removed by vortexing with 150 µl of MPC Protein Precipitation Reagent and following centrifugation for 10 minutes at 17,000× g. DNA was recovered with 500 µl isopropanol and centrifugation at 17,000× g for 10 minutes. DNA was purified by RNase A treatment for 60 minutes at 37°C followed by phenol:chloroform extraction and ethanol precipitation. DNA yield and quality was determined by spectrophotometry. 2 µg of genomic DNA were used for library preparation: DNA was fragmented to 150–500 bp using Covaris shearing and processed with the TruSeq DNA Sample Prep Kit (Illumina) according to instructions, purification steps were performed with Agencourt AMPureXP magnetic particles (Beckham Coulter) on a magnetic stand (AmBio). Whole genomes were sequenced on an Illumina HiSeq2000 at the MRC Clinical Science Centre, Imperial College London (UK).

### Illumina Sequence Analysis

Alignment and SNP calling parameters were initially optimized. The nuclear and mitochondrial genome sequences and feature files for *C. gattii* isolate R265 (VGII) were downloaded from http://www.broadinstitute.org/ (GenBank project accession number AAFP01000000). Illumina reads were aligned to the genome sequence using Burrows-Wheeler Aligner (BWA) v0.5.9 [81] with default parameters and converted to pileup format using Samtools v.0.1.18 [82]. To act as a control for sequencing, alignment and SNP calling, we resequenced the reference strain R265. We used a False Discovery (FDR) approach [83] to test our SNP-calling method, which we set at a minimum required depth of four reads, with 90% disagreeing from the reference base and agreeing with each other. First, we randomly modified 63,193 and 698,535 nucleotides within the reference sequence, corresponding with the maximum number of SNPs identified within the VGII group and within any of our *C. gattii* isolates, respectively. We aligned the reads of R265 to these two altered genome sequences and called SNPs using our chosen parameters. We identified 99.32% and 99.26% of true positives, whilst only calling 3553 (5.6%) and 3363 (0.48%) false positives or genuine discrepancies with the reference sequence respectively. For analysis we used all SNPs that were covered by ≥4 reads in all isolates, leaving a total 740 mitochondrial sites. To ensure that these sites could not have been heterogeneous in the progeny sequence we examined the allele frequencies at each variant site and found that each site had greater than 95% agreement with the called SNP. We simulated 100 bp reads with 0.01% uniform error for 200× coverage from each parental mitochondrial genotype and used them in a single combined BWA alignment and SNPs calling protocol as before. We detected only seven sites that show shared differences from the reference sequence of R265. The other SNPs were not detected because they did not reach the 90% agreement threshold.

### *De Novo* Assemblies of Mitochondrial Genomes

Two to four million 100 bp Illumina paired end reads were assembled with velvet (version 1.2.08) [84]. Redundant assembly runs with varying k-mer lengths were performed for each strain, and each time resulted in identical circular contigs, which only differed in lengths of overlapping ends.

### Chromosomal Ploidy Analysis of VGII x VGIII Progeny

Supercontigs of *C. gattii* R265 were obtained from Broad Institute (http://www.broadinstitute.org), and grouped in chromosomal context by alignment to *C. gattii* WM276 chromosomes [77] with MUMmer (version 3.23) [85], [86]. The resulting tiled R265 chromosomes served as reference in downstream analyses.

Ploidy of EJB and SP strains was assessed by read coverage and allele frequencies at variant sites. After mapping with Bowtie 2 (version 2.1.0) [87], read coverages were calculated and plotted with CNV-seq [88]. *C. gattii* WM276 was used as mapping genome, a theoretical VGII x VGIII diploid of equally pooled R265, B4546 reads as reference, and R265 x B4546 progeny (Progeny and SP strains) reads as testing samples, respectively.

Allele frequencies of variant sites in Progeny and SP progeny were calculated after Bowtie 2 read mapping to R265 chromosomes, and variant calling with samtools/bcftools from the samtools package (version 0.1.18 (r982:295)) [82] Allele frequencies were extracted from DP4 fields of VCF output, and nucleotide variant ratios calculated for each position by division of reads depth/number of variant bases. A separate mapping of R265 reads was performed as control, and observed positions removed as background noise from Progeny and SP variant calls. Additional details on the ploidy analyses are available in the supplementary information (Text S1).

### Alignment of Mitochondrial Genomes

To assess mitochondrial recombination, genomes of CBS10090 and NIH312 were compared to CBS10090 x NIH312 progeny strain Progeny 5. Sequences were aligned, and corresponding regions visualized with Progressive Mauve (version 2.3.1) [89]. Additional details on the analysis of mitochondrial genomes are available in the supplementary information (Text S1).

## Supporting Information

Figure S1 Ploidy analysis in B4546 x R265 progeny. **A**) Copy number variations in individual chromosomes were examined by read coverage plots with CNV-seq. Illumina reads of testing strains (Progeny, restored haploid SP progeny of R265 and B4546) were mapped to WM276, and read coverage compared to a reference sample of equally pooled reads of R265 and B4546. Y-axis: log2 coverage ratios of individual testing strains and the reference. Log2 ratios of approx. 0 correspond to diploid chromosomes, ratios of approx. 0.5 to triploid chromosomes respectively. X-axis: the 14 chromosomes of each strain are represented in different colors. **B**) Mean chromosomal allele frequencies at variant sites in R265 x B4546 progeny. Variant base ratios have been calculated by division of the number of variant bases/read depth at each position. Columns contain mean variant ratios per chromosomes in EJB and SP strains. “na” indicates ratios in chromosomes with less than 100 variants, which were presumably detected due to sequencing errors.
(TIF)

Figure S2 Correlation analysis of intracellular proliferation and yeast mitochondrial tubularisation within macrophages for crosses. **A**) B4546 x R265, **B**) CBS10090 x NIH312, **C**) YL9 x 97/433, **D**) AIg289 x JF101, **E**) AIg254 x JF109, **F**) CBS1930 x R265, **G**) LA584 x R265 and **H**) all parental strains and progeny.
(TIF)

Table S1 Genbank accession numbers for MLST alleles used and previously published for isolates R265, CBS10090, NIH312 and B4546.
(DOCX)

Table S2 MLST primers used for analysis of outgroup cross progeny.
(DOCX)

Text S1 Additional information on ploidy and mitochondrial genome analyses.
(DOCX)
