# Supplementary figures and images for: Transmission of Hypervirulence Traits via Sexual Reproduction within and between Lineages of the Human Fungal Pathogen Cryptococcus gattii
Source: PLoS Genet. 2013 Sep 5;9(9):e1003771. doi: 10.1371/journal.pgen.1003771 (PMC3764205; doi:10.1371/journal.pgen.1003771)

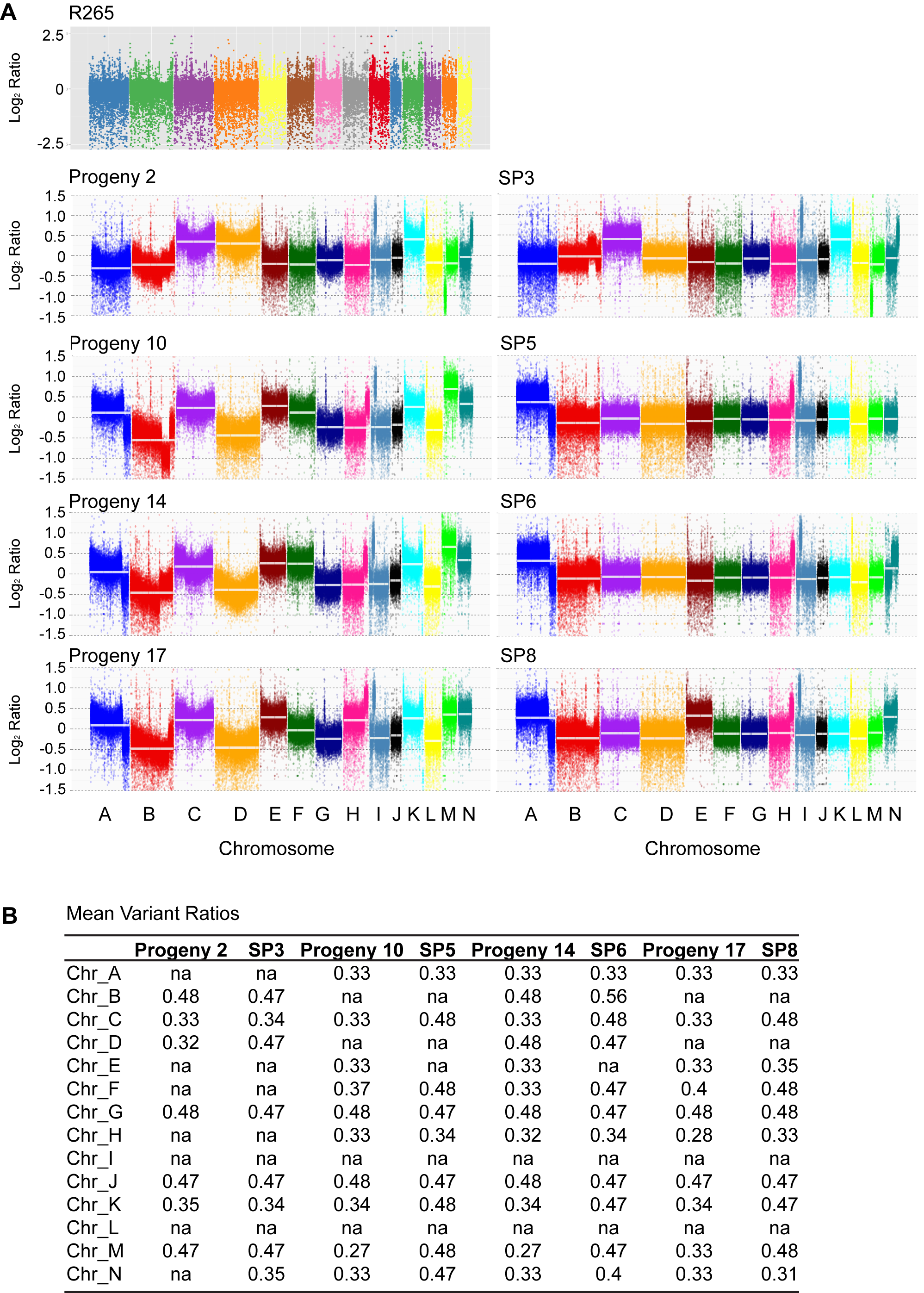

Supplement: Figure S1 — Ploidy analysis in B4546 x R265 progeny. A) Copy number variations in individual chromosomes were examined by read coverage plots with CNV-seq. Illumina reads of testing strains (Progeny, restored haploid SP progeny of R265 and B4546) were mapped to WM276, and read coverage compared to a reference sample of equally pooled reads of R265 and B4546. Y-axis: log2 coverage ratios of individual testing strains and the reference. Log2 ratios of approx. 0 correspond to diploid chromosomes, ratios of approx. 0.5 to triploid chromosomes respectively. X-axis: the 14 chromosomes of each strain are represented in different colors. B) Mean chromosomal allele frequencies at variant sites in R265 x B4546 progeny. Variant base ratios have been calculated by division of the number of variant bases/read depth at each position. Columns contain mean variant ratios per chromosomes in EJB and SP strains. “na” indicates ratios in chromosomes with less than 100 variants, which were presumably detected due to sequencing errors. (TIF) [file pgen.1003771.s001.tif]

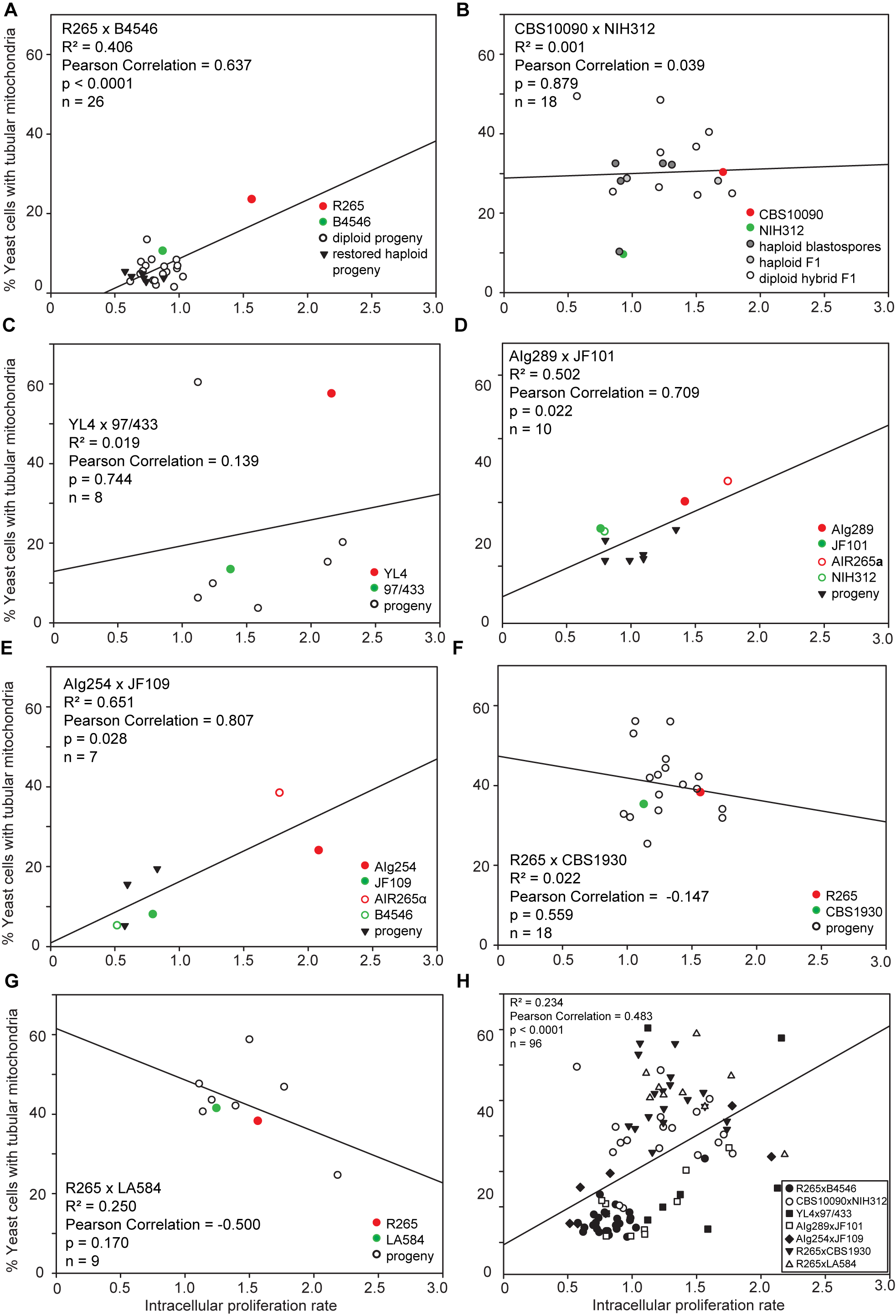

Supplement: Figure S2 — Correlation analysis of intracellular proliferation and yeast mitochondrial tubularisation within macrophages for crosses. A) B4546 x R265, B) CBS10090 x NIH312, C) YL9 x 97/433, D) AIg289 x JF101, E) AIg254 x JF109, F) CBS1930 x R265, G) LA584 x R265 and H) all parental strains and progeny. (TIF) [file pgen.1003771.s002.tif]
