# Supplementary material for: Transmission of Hypervirulence Traits via Sexual Reproduction within and between Lineages of the Human Fungal Pathogen Cryptococcus gattii
Source: PLoS Genet. 2013 Sep 5;9(9):e1003771. doi: 10.1371/journal.pgen.1003771 (PMC3764205; doi:10.1371/journal.pgen.1003771)
Supplement: Table S1 — Genbank accession numbers for MLST alleles used and previously published for isolates R265, CBS10090, NIH312 and B4546. (DOCX) [file pgen.1003771.s003.docx]

| **GenBank accession number** | **Submission Details** |
| --- | --- |
| DQ096307 | MLST allele SXI1α_16 |
| DQ096308 | MLST allele SXI1α_18 |
| DQ096310 | MLST allele SXI2**a**_4 |
| DQ096314 | MLST allele IGS_4 |
| DQ096327 | MLST allele IGS_18 |
| GU299206 | MLST allele IGS_31 |
| DQ096360 | MLST allele TEF_3 |
| DQ096364 | MLST allele TEF_7 |
| DQ096367 | MLST allele TEF_10 |
| DQ096377 | MLST allele GPD_1 |
| DQ096383 | MLST allele GPD_7 |
| DQ096385 | MLST allele GPD_9 |
| GU299205 | MLST allele GPD_27 |
| DQ096398 | MLST allele LAC1_2 |
| DQ096400 | MLST allele LAC1_4 |
| DQ096405 | MLST allele LAC1_9 |
| DQ096416 | MLST allele CAP10_1 |
| DQ096421 | MLST allele CAP10_6 |
| DQ096343 | MLST allele PLB1_1 |
| DQ096346 | MLST allele PLB1_4 |
| DQ198349 | MLST allele PLB1_23 |
| DQ096334 | MLST allele MPD1_5 |
| DQ096337 | MLST allele MPD1_8 |
