# Supplementary material for: Transmission of Hypervirulence Traits via Sexual Reproduction within and between Lineages of the Human Fungal Pathogen Cryptococcus gattii
Source: PLoS Genet. 2013 Sep 5;9(9):e1003771. doi: 10.1371/journal.pgen.1003771 (PMC3764205; doi:10.1371/journal.pgen.1003771)
Supplement: Table S2 — MLST primers used for analysis of outgroup cross progeny. (DOCX) [file pgen.1003771.s004.docx]

| **Primer Name** | **Primer Sequence** | **Marker Amplified** |
| --- | --- | --- |
| JOHE10451 | TACATCACCGGTCATATCTGC | *SXI1*α |
| JOHE10452 | CTGGAGAAGCGCCTCACTGGA | *SXI1*α |
| JOHE14115 | AGGGTACGTTTGAGGCCAGTT | *SXI1*α alternative |
| JOHE14116 | GAAAGCGTTGGCAAGGAATGA | *SXI1*α alternative |
| JOHE10453 | TGATCGCACGAGCCAAATCCC | *SXI2***a** |
| JOHE10454 | GGCTTCCTGACAACACTTCTA | *SXI2***a** |
| YL001 | TGTCTTCTGACACCCAGTCG | *SXI2***a** alternative |
| YL002 | TGTCTTCTGACACCCAGTCG | *SXI2***a** alternative |
| JOHE14408 | ATCCTTTGCAGACGACTTGA | *IGS* |
| JOHE14409 | GTGATCAGTGCATTGCATGA | *IGS* |
| JOHE14976 | GCACGCTCTTCTCGCCTTCAC | *TEF1* |
| JOHE14977 | GTAGTCGGCGTAGGTCTCAAC | *TEF1* |
| JOHE14968 | CCACCGAACCCTTCTAGGATA | *GPD1* |
| JOHE14969 | CTTCTTGGCACCTCCCTTGAG | *GPD1* |
| JOHE14970 | AACATGTTCCCTGGGCCTGTG | *LAC1* |
| JOHE14971 | AACATGTTCCCTGGGCCTGTG | *LAC1* |
| JOHE14386 | CCGGAACTGACCACTTCATC | *CAP10* |
| JOHE14387 | GCCCACTCAAGACACAACCT | *CAP10* |
| JOHE14974 | CTCTCATTGTTCGCCGCTACT | *PLB1* |
| JOHE14975 | GGAAGCCGAGGTCTGATTTGG | *PLB1* |
| JOHE14972 | TGCCCTGGATCCTAATGCTCT | *MPD1* |
| JOHE14973 | ACCCAGACTGCCGCTGTCGTC | *MPD1* |
