# Supplementary material for: Transmission of Hypervirulence Traits via Sexual Reproduction within and between Lineages of the Human Fungal Pathogen Cryptococcus gattii
Source: PLoS Genet. 2013 Sep 5;9(9):e1003771. doi: 10.1371/journal.pgen.1003771 (PMC3764205; doi:10.1371/journal.pgen.1003771)
Supplement: Text S1 — Additional information on ploidy and mitochondrial genome analyses. (DOCX) [file pgen.1003771.s005.docx]

Ploidy analysis

Variations in chromosomal copy numbers in VGII x VGIII progeny strains were assessed by combined analysis of read coverage and nucleotide variant frequency. Read coverage calculations were based on normalized log2 coverage ratios of reference (R265 x B4546) and testing read samples (diploid and haploid restored progeny), which have been independently mapped to the VGI reference genome of *C. gattii* WM276. Coverage plots (Figure S1A) revealed two abundant log2 ratio levels at 0.0, corresponding to the euploid (2n) testing sample, and approximately 0.5, indicating triploid chromosomes, with exception of chromosome M in Progeny 10 and 14, which displayed significantly higher read coverages, thus indicating 4n. To verify implications from read coverage analyses, we examined mean allele frequencies at variant sites over individual chromosomes (Figure S1B). Observed mean variant ratios of approx. 0.5, resulting from 50% variant and 50% reference bases, and therefore indicated diploid chromosomes, where haploid copies have been equally inherited from both parental strains (R265, B4546). Likewise triploidy was revealed by mean ratios of approx. 0.33, resulting from two chromosomal copies of R265, and one copy of B4546, respectively. Interestingly ratios of 0.66, which would correspond to triploidy, with two chromosomal copies from B4546 were not observed. The presumably tetraploid chromosome M in Progeny 10 and 14 each yielded variant ratios of 0.27, which is close to the theoretical value of 0.25 (assuming 3x R265, 1x B4546). In several chromosomes less then 100 variants were detected, which most likely occurred due to sequencing errors and therefore indicat homozygous copies of R265 chromosomes. Chromosomes I and L showed this characteristic in all progeny strains. In summary, our combined analyses identified several aneuploid chromosomes in diploid and haploid restored progeny (Figure S1).

Mitochondrial genome analysis

De novo assemblies revealed that mitochondrial genomes of VGII and VGIII strains differ significantly in size. Genome sizes in examined VGII strains varied between 28-30 kb (R265: 31198 bp, CBS10090: 28993 bp, CBS1930: 31214 bp), while VGIII genomes are of almost double size (B4546: 57471 bp, NIH312: 58233 bp). Alignment of VGII and VGIII genome sequences revealed extensive repetitive regions, disrupting conserved regions, which are shared between strains. Assembled mitochondrial genomes of Progeny from the outgroup cross B4546xR265 and restored haploid SP strains were 100% identical to B4546, indicating that only VGIII mitochondria were inherited to progeny of R265 and B4546 crosses, and that furthermore no mitochondrial recombination occurred. In contrast the mitochondrial genome of Progeny 5, progeny of CBS10090 x NIH312, was not inherited by either parent, which became obvious by its size of 36283 bp. The genome furthermore revealed DNA regions, which are unique to either parental strain, indicating extensive recombination (Figure 10B). In addition, three mitochondrial markers were used to assess the inheritance of mitochondrial DNA in the VGII x VGII crosses. In four examples (Figure 10C), recombinant classes were observed. This finding supports previous evidence for mitochondrial recombination in *C. neoformans* congenic serotype A strains KN99**a** and KN99α, which were derived from H99alpha, but have a recombinant mitochondrial genome that resulted from the backcross scheme and is derived from both the **a** parent and a recombination event [51,27] and evidence from AFLP analysis of *C. gattii* isolates [52].
